# Overexpression of *OsF*_*3*_*H* modulates WBPH stress by alteration of phenylpropanoid pathway at a transcriptomic and metabolomic level in *Oryza sativa*

**DOI:** 10.1038/s41598-020-71661-z

**Published:** 2020-09-07

**Authors:** Rahmatullah Jan, Muhammad Aqil Khan, Sajjad Asaf, In-Jung Lee, Kyung-Min Kim

**Affiliations:** 1 Division of Plant Biosciences, School of Applied Biosciences, College of Agriculture and Life Science, Kyungpook National University, 80 Dahak-ro, Buk-gu, Daegu, 41566 Republic of Korea; 2 Natural and Medical Science Research Center, University of Nizwa, Nizwa, 616 Oman

**Keywords:** Agricultural genetics, Gene expression, Gene regulation, Plant breeding, Molecular biology

## Abstract

The whitebacked planthopper (WBPH), has become a devastating pest for rice crops, causes serious yield losses each year, and urgently needs biological control. Here, we developed a WBPH-resistant rice cultivar by overexpressing the OsF3H gene. A genetic functional analysis of the OsF3H gene confirmed its role in facilitating flavonoid contents and have indicated that the expression of the OsF3H gene is involved in regulation of the downstream genes (OsDFR and OsFLS) of the flavonoid pathway and genes (OsSLR1 and OsWRKY13) involved in other physiological pathways. OxF3H (OsF3H transgenic) plants accumulated significant amounts of the flavonols kaempferol (Kr) and quercetin (Qu) and the anthocyanins delphinidin and cyanidin, compared to the wild type, in response to the stress induced by WBPH. Similarly, OsF3H-related proteins were significantly expressed in OxF3H lines after WBPH infestation. The present study, indicated that the regulation of JA in OxF3H plants was suppressed due the overexpression of the OsF3H gene, which induced the expression of downstream genes related to anthocyanin. Similarly, the OsWRKY13 transcriptional factor was significantly suppressed in OxF3H plants during WBPH infestation. Exogenous application of Kr and Qu increased the survival rates of susceptible TN1 lines in response to WBPH, while decreased the survival rate of first instar WBPHs, indicating that both flavonols exhibit pesticide activity. Phenotypic demonstration also affirms that OxF3H plants show strong resistance to WBPH compared with wild type. Collectively, our result suggested that OsF3H overexpression led to the up-regulation of defense related genes and enhanced rice resistance to WBPH infestation.

## Introduction

Rice (*Oryza sativa *L.) is an important food staple for 50% of the world's population, with particular importance in Asia. It is cultivated in approximately 114 countries and is a primary source of income and employment for more than 100 million households in Asia and Africa^1^. A recent survey showed that rice production in developing countries is lower than consumption due to population increases, constituting a need to increase production by 70% over the next 30 years to cover the deficit^2^. Rice flourishes in warm and humid climates, which are also conducive to pest proliferation. Pest infection of rice crops can ultimately decrease yield, and intercropping practice utilization, tillering variety cultivation, and the widespread use of synthetic fertilizers promotes pest development^3^. Control measurements are not in place when pests are initially introduced to a new locality, but can become out of control at later stages, severely damaging crops. The white-backed planthopper (WBPH), *Sogatella furcifera* (Horvath), is a serious insect pest. The first outbreak of WBPH was reported in India in 1966, and rapidly spread to China, Korea, and Japan^4–6^. WBPH initially spread to Korea and Japan from China. China loses 10–20% of its rice yield every year due to WBPH infestation^5^. In Korea, WBPH is still considered a minor pest and significant destruction has not yet been reported. However, if pest control was not implemented, it could have become a serious problem.

WBPH damages plants through cell sap sucking and viral transmissions, like grassy stunt and rugged stunt^3^. Until 1986, WBPH was not considered a viral vector, but rather that it caused nutrient deficiency by sucking sap, which could kill the plant^7^. However, in 2001 it was reported that WBPH transmitted a virus, namely the southern rice black-streaked dwarf virus, causing outbreaks in hybrid rice in China and Vietnam^8^. Leafhoppers drill into plant tissues using fine stylets, making tiny punctures. After penetration, the insects assess the physiochemical properties around the tip of the stylet, sensing high sugar concentrations^9^. Plant phloem contains a complex mixture of organic and inorganic substances, with amino acids and sugar predominating^10^. Previous reports have demonstrated that phloem sap sucking pests feed on its sugar content (sucrose, glucose and fructose), as a source of energy^11^. Given the destructive potential of WBPH in rice, it is necessary to produce resistant variants to compensate for this.

Plants are frequently exposed to pathogens, viruses, and herbivores, and as a defensive reaction they regulate secondary metabolites to deal with biotic stress, usually induced by herbivores^12,13^. Secondary metabolites, including flavonoids, play a significant role in plant adaptation to environmental stress, including pest-induced stress^14,15^. The demand for pesticides is growing, and scientists have focused on flavonoids for pesticides, as they prevent pest larval growth through enzymes inhibition^16^. Off all the flavonoids, Kr and Qu are the most important pest repellent molecules, and reports show that kaempferol-3,7-diglucoside can be used as a *Mamestraconfigurata* deterrent, and Qu can be used for *Spodopteralitura* larvae^17,18^. Kr and Qu are strong antioxidants and are highly efficient free radical scavengers. Kr contains a 3-OH group, while Qu contains 3 and 5 OH groups in the C and A rings, respectively, with the capacity to donate the most electrons^19,20^. Secondary metabolites (flavonoids) are positively regulated during biotic stress due to pathogenic or fungal attacks^21^. Reactive oxygen species (ROS) are produced in response to pathogenic attacks, which harms plant tissue, and plants prevent the accumulation of ROS through antioxidant activity as a defense mechanism^22–24^. Previous reports show that Qu deposits in plant epidermal and mesophyll tissues protect infected tissues from oxidative stress mitigated by high light intensity^25^.

Here, we aimed to develop WBPH-resistant variants, as development and cultivation of resistant varieties is a secure, economically reasonable, and attractive method of pest control. The use of transgenic techniques is a feasible method for inserting exogenous genes into plant genomes to developed new varieties, instead of conventional, time-consuming breeding methods^26,27^. We focused on the development of the flavonoid biosynthesis pathway in rice, especially Kr, Qu, and anthocyanin, through *OxF*_*3*_*H* overexpression. The *OxF*_*3*_*H* gene encodes flavonone-3-hydroxylase, which regulates dihydro-kaempferol and dihydro-quercetin biosynthesis, which increases Kr and Qu biosynthesis. *F*_*3*_*H* expression also enhances anthocyanin biosynthesis^28,29^. Previous reports have shown that *CHI* and *F*_*3*_*H* expression enhances anthocyanin biosynthesis^30^. Bogs et al.^31^ reported that *F*_*3*_*H* converts flavanones to dihydroflavonols, which further upregulates anthocyanin biosynthesis. A literature review showed that the *WARKY13* transcription factor regulates chalcone synthase (*CHS*), which is the main enzyme involved in flavonoid biosynthesis in the PAL pathway^32^. Genome expression profiling showed that *WARKY13* also induced *F*_*3*_*H* and a zinc-finger protein, which is homologous to *TT* of *Arabidopsis,* both of which are downstream of *CHS*^33^. *WARKY13*, *F*_*3*_*H*, anthocyanin, and jasmonic acid are interlinked in the defense mechanism against biotic stress. Anthocyanin is related to jasmonic acid, which is an essential signaling phytohormone which is positively regulated during pathogenic attacks and stress^34,35^. JA enhances anthocyanin biosynthesis and acts as a strong regulator of elicitor signals to increase the biosynthesis of secondary metabolites, and actively participates pathogen- and wound response^36–38^. Here, we evaluated the roles of *WARKY13*, JA, anthocyanin, Kr, and Qu in the *OxF*_*3*_*H* rice strain, during stress induced by WBPH.

## Materials and methods

### Plasmid construction and cloning via the gateway method

The Cheongcheong cultivar was provided by the Plant Molecular Breeding lab, Kyungpook National University, and was used for RNA isolation and *OsF*_*3*_*H* gene amplification. Before sowing, seeds were soaked in an incubator for 3 days at 30 °C, and the water changed each day. Germinated seeds were transplanted to pots, kept in the dark for 3 days, and then transferred to greenhouses. Total RNA was isolated from young leaves of 14-day old rice seedlings, using RNeasy Plant Mini Kits from Qiagen. Standard cDNA was synthesized from total RNA using qPCRBIO cDNA Synthesis Kits, from PCRBIOSYSTEMS, according to the manufacturer’s instructions. The full length ORF region of *OsF*_*3*_*H* (483 bp) was amplified by PCR, using gene-specific primers, with four additional nucleotides “CACC” attached to the 5′ end of the forward primer (Supplemental Table S1). The gateway cloning system was followed. Entry clones were generated by inserting template DNA into pENTR/D-TOPO cloning vectors, following the manufacturer’s instructions [for details, see the user manual for the pENTR Directional TOPO cloning kit (Invitrogen)]. The TOPO cloning reaction was transformed into DH5α cells using heat shock, and spread on LB media containing kanamycin as a selection marker. Plasmids were isolated from selected colonies and grown overnight in liquid LB media containing kanamycin, using QIAprep Spin Miniprep Kits from Qiagen. Isolated plasmids were double digested with Not1 and Asc1 restriction enzymes, checked on gels (Supplemental Figure S1A), and confirmed by sequencing. For overexpression vector construction, entry clone fragments were inserted into BamH1 and Xho1 sites of the destination vector, pSB11, under the control of a 35S promoter, through LR reaction using Gateway LR Clonase enzyme mix kits (Invitrogen). The constructs were transformed into *Agrobacterium* cells LBA4404 (Takara) via heat shock and spread on spectinomycin-containing LB media. Plasmids were isolated and double digested with BamH1 and Xho1 restriction enzymes, and the ligation and transformation confirmed by running the digested reactions on gels (Supplemental Figure S1B).

### Generation of transgenic rice

A line of *OsF*_*3*_*H* (*OxF*_*3*_*H*) overexpression rice was developed through callus culture, according to the method described by Sahoo et al.^39^, with minor modifications. For callus induction, good quality mature Nagdong rice seeds were selected, gently dehulled, and sterilized with 70% ethanol for 5 min with continuous shaking, and then washed three times with double distilled water. The seeds were then sterilized with 3% sodium hypochlorite for 10 min with shaking, rinsed with sterilized water three times, and dried for 1 h on a clean bench. Dried seeds were inoculated into callus induction medium, with 10–15 seeds per plate, and placed in dark conditions for 12 days. All medium types and compositions are presented in Supplemental Table S2. After growing for 12 days, calluses were pre-cultured into small pieces of calli and inoculated for 3 days in callus induction media under dark conditions. At the same time, *Agrobacterium* strain LBA4404 harboring the pSB11 plasmid containing the full length *OsF*_*3*_*H* gene was cultured by selecting single colonies from the transformed plates, inoculating them in 5 ml LB medium containing 50 mg/l spectinomycin, and incubation for 16–18 h in a shaking incubator at 28 °C. Further cultures were prepared in autoclaved baffled flasks containing 100 ml medium, under the same conditions, and the cells harvested when O.D. reached 600. The pelleted cells were resuspended in MS medium fortified with acetosyringone, and the calli were immersed in the suspension for 30 min with continuous shaking. Excess *Agrobacterium* cells were removed by drying for 30 min on sterilized filter paper and then inoculated into co-cultivation medium for 3 days under dark conditions. Excessive *Agrobacterium* growth was controlled by washing three times with 500 mg/l carbenicillin, drying for 30 min, and inoculating into first selection medium containing 50 mg/l spectinomycin under light conditions (16/8 photoperiod) for 12 days. The calli were transferred to second selection medium for 10 days, followed by third selection medium for 5 days in light conditions (16/8 photoperiod). Black and brown calli were removed and creamish proliferated spectinomycin-resistant calli were transferred to the regeneration medium and incubated for 10 days under dark conditions at 27 °C during the first phase. In the second phase, the calli were transferred to new identical medium and placed in light conditions until plantlets developed. In the third phase, the plantlets were placed in test tubes on the same medium for root-development. After 20 days growth in test tubes the plants were properly developed and transferred to soil pots.

### Test of ***OxF***_***3***_***H*** plants’ resistance to WBPH and WBPH rearing

The experiment aimed to determine the *OxF*_*3*_*H* plant resistance to WBPH. *OxF*_*3*_*H* and wild-type plant seeds were sterilized with fungicides overnight, washed three times, and incubated for 3 days at 30 °C. Approximately 20 germinated seeds of each (wild-type and *OxF*_*3*_*H)* were transferred to autoclaved soil contained in individual pots, in multiples of three, and grown in a growth chamber for one week. Seedlings were transferred to a greenhouse and about 200 male and female WBPH (2nd and 3rd instar) were separately introduced to both *OxF*_*3*_*H* and wild-type plants after three weeks of growth, at a ratio of 3 WBPH per plant. Before introductions, WBPH were kept in a beaker, starved with wet tissue for 2 h. Phenotypic evaluation, including the number of infected plants, was counted after 10, 50 and 70 days, and the plants were scored as resistant or susceptible, based on infection vs non-infection. Plants were considered infected based on observed symptoms (brownish spots of wounds). Plants with no symptoms were considered non-infected. Samples for RNA isolation, hormonal regulation, and proteomic expression were collected according to further experimental design. WBPH was provided by the National Institute of Crop Science, Rural Development Administration, Korea. A population of WBPH was maintained continuously in the insectarium on susceptible TN1 rice, at Kyungpook National University, under ideal environmental conditions (temperature 27 ± 1 °C, humidity 60–70%, and light cycle 16-h) following the methods described by Vicheka et al.^40^ and Yun et al.^41^.

### Quantitative real-time PCR

Total RNA was isolated from 10 leaves of 10 wild-type and *OxF*_*3*_*H* plants infested with WBPH in triplicate after two, 12, and 24 h of WBPH infestation. First, standard cDNA was synthesized using qPCRBIO cDNA Synthesis Kits from PCRBIOSYSTEMS, following the manufacturer’s instructions. Real-time PCR was performed using qPCRBIO SYBR Green Kits from PCRBIOSYSTEM, following the manufacturer’s instructions. Real-time PCR was conducted using an Illumina Eco Real-Time PCR System (Singapore), following the manufacturer’s procedures, to relatively quantify the expression levels of flavanone 3-hydroxylase (*OxF*_*3*_*H*), flavonol Synthase (*OsFLS*), dihydroflavonol 4-reductase (*OsDFR*), *OsWRKY13*, and *OsSLR1* (Della protein). The primers are listed in Supplemental Table S1. To standardize the level of expression of each gene, actin was used for each reaction and the expression level was calculated in wild plants infested with WBPH relative to *OxF*_*3*_*H* infested with WBPH. The reaction was performed in a 20 µl volume containing 7 µl ddH_2_O, 1 µl primer, 10 µl SYBR green, and 1 µl cDNA, with each reaction repeated three times.

### Western blot analysis

Western blotting was performed to assess protein expression in the transgenic line, following the method optimized by Hao et al.^42^, with slight modifications. Proteins of the *OxF*_*3*_*H* line were collected at 2, 12, 24, and 36 h after WBPH infestation. Total protein was isolated with the 10 ml TCA/Acetone (10% Trichloroacetic acid (TCA); 0.07% β-ME in Acetone P.A.) method proposed by Xu et al.^43^. Equal amounts of protein were boiled for 5 min and separated on 10% SDS–PAGE at 100v for 150 min, and then transferred to a NC membrane (Whatman Japan) by a semi-dry method running for 90 min at 19v, using a Trans-Blot DS semi-dry transfer cell (Bio Rad). The membrane was blotted in TBST (0.1% Tween 20 in TBS) and 5% non-fat dry milk (w/v) for 2 h at room temperature. Proteins were further blotted with primary rabbit anti-F3H antibodies in 5% non-fat dry milk (w/v) and TBST overnight at 4 °C, and rinsed three times for 10 min in TBST solution. The membranes were then incubated in Gt anti-Ms IgG (H + L) secondary antibodies (Invitrogen USA), at a dilution of 1:1,000, for 2 h at room temperature, and rinsed three times for 10 min in TBST solution. The blot was developed with Amersham ECL (GE Healthcare UK), and protein bands were exposed on X-ray film. Western blotting and quantification analyses were performed in at least two biological replications (Supplemental Figure S2).

### In situ detection of kaempferol and quercetin in plant tissues by DPBA staining

Albino wild-type and *OxF*_*3*_*H* rice seedlings (Supplemental Figure S3) were grown on 3 ppm Norflurazon (Sigma-Aldrich) subjected filter paper in petri plates, as norflurazon is an herbicide that eliminates autofluorescence chlorophyll and carotenoid. After growing for 10 days, approximately 10 WBPH (male and female) were inoculated into each plate (5 plants) and samples were collected for DPBA staining when symptoms appeared^44^. Targeted areas of leaves, stems, and roots were collected for staining. Diphenylboric acid-2-aminoethyl ester (DPBA) solution was prepared by mixing 0.25% (0.25 g) DPBA and 200 µl of 0.02% Triton X-100 (v/v) (Sigma-Aldrich) in distilled water, at a 100 ml final volume. Collected samples were incubated with 0.25% staining solution in a vacuum for 5 min, with incubation times dependent on the tissue type. For example, a whole plant needs 1- to 2 h incubation. A Confocal Laser Scanning microscope model Carl Zeiss (LSM700) was used to detect the fluorescence of Kr, Qu, and naringenin. A FITC filter (suppression LP 488 nm) allowed visualization of Kr (green), while an R-PE filter (suppression LP 488 nm) and Rhodamine (suppression LP 555 nm) visualized Qu (orange) and naringenin (red), respectively.

### Flavonol and anthocyanin extraction and LCMS–MS analysis

To determine relative anthocyanin (cyanidin and delphinidin) quantification in *OxF*_*3*_*H* and wild-type subjected to WBPH stress, total anthocyanin was extracted from 20 plant shoots of wild-type and *OxF*_*3*_*H* controls, and wild-type and *OxF*_*3*_*H* infested with WBPH, in triplicate using the method described by Neff and Chory^45^, with slight modifications. Shoots of selected plants were ground into fine powders in liquid nitrogen, with a pre-cooled mortar and pestle. To extract anthocyanins, 5 g powder was homogenized with 50 ml methanol (1% HCl), with continuous shaking for 6 h at 4 °C. Each sample was vortexed, filtered using filter paper, the remaining residues extracted with 30 ml methanol (1% HCl), after which the process was repeated. The crude extracts were further diluted with a rotary evaporator to 2 ml at 30 °C, and further dried in a heating block at 60 °C overnight. To extract flavonols (kaempferol and quercetin), 3 g of the remaining ground samples were homogenized in 30 ml of methanol mixture (MeOH:H_2_O:HCl = 79:20:1, v/v/v), and shaken for 6 h in a shaking incubator. Extracts were filtered and the same processes were followed for dilution and drying as for anthocyanins. Both the dried anthocyanin and flavonol samples were dissolved in 1 ml HPLC grade ethanol. Reference standards for liquid chromatography–mass spectrometry (LC–MS) were prepared by dissolving 1 mg of each standard sample in 1 ml ethanol. For quantification of sample concentrations, a TSQ vantage triple quadrupole mass spectrometer consisting of a HESI-II Spray source coupled to a Shimadzu Prominence UFLC system (Kyoto, Japan) incorporating a DGU-20A_5_ degasser, LC-20AD Pump, SIL-20A autosampler, and CTO-20A column oven, was used for analysis. Shim-pack GIS C18 columns (150 × 3.0 mm, 3 µM) were used to separate the analytes in the samples. The mobile phase contained 100% water with 0.1% formic acid (mobile phase A), and ACN with 0.1% formic acid (mobile phase B), at a flow rate of 0.50 mL/min at 40 °C. Gradient conditions were as follows: 20% of B for 0–0.25 min, gradually increased from 20–80% of B at 0.25–2 min, 80% of B at 2–7 min, 80–20% of B at 7–7.5 min and 20% of B at 7.5–10 min. The MS operating conditions were as follows: electrospray ionization in negative mode, 3,000 V and 4,000 V in positive mode, capillary temperature at 350 °C; vaporizer temperature at 300 °C; sheath gas pressure at 35 Arb; auxiliary gas pressure at 10 Arb. Finally, all data were analyzed using Xcalibur software (Thermo Fisher Scientific Inc., USA).

### JA quantification

The endogenous JA contents were extracted from both wild-type and *OxF*_*3*_*H* plants after inoculation with WBPH (discussed in “Test of OxF3H plants’ resistance to WBPH and WBPH rearing”). Samples were collected at 2-, 12-, and 24 h, and approximately eight to ten plant leaves were randomly collected in triplicate, following the protocol described by Bilal et al.^46^ with slight modifications. Approximately 1 g freeze-dried leaves of wild-type and *OxF*_*3*_*H* plants was ground into a fine powder in liquid nitrogen, with a chilled mortar and pestle. About 0.3 g of the ground sample was mixed in an extraction mixture of acetone and 50 mM citric acid (70:30, v/v), and kept overnight at a low temperature to evaporate highly volatile solvents. The remaining crude extract was filtered through Whatman filter paper and further extracted with 10 ml diethyl ether for 5 repetitions. The extract was then loaded on the solid phase extraction cartridge (500 mg sorbent, aminopropyl), and the cartridge washed with 8.0 ml trichloromethane and 2-propanol (3:1, v/v). The extracted JA and standard were diluted with 10 ml diethyl ether and acetic acid (97:3, v/v). Samples were evaporated, the residue esterified with diazomethane, and the volume adjusted to 50 ml with dichloromethane. The purified extract was then subjected to GC–MS (6890N network GC system and the 5,973 network mass selective detector; Agilent Technologies, Palo Alto, CA, United States). The ion portion was checked at m/z D 83amu, analogous to the JA base peaks. The quantification of endogenous JA was measured from the peak areas compared with the relevant standards.

### Exogenous application of kaempferol and quercetin into WBPH inoculated plants

A separate experiment was conducted to elucidate the effect of exogenous application of Kr and Qu on WBPH. TN1 susceptible lines were used and experiments were designed using five treatments in three groups: (1) control; (2) 3 ppm Kr sprayed and supplemented into soil; and (3) 3 ppm Qu sprayed and supplemented into soil, and all three groups were placed in a separate insectarium. All treatments were grown in autoclaved soil and magenta boxes, under favorable conditions of 27 °C and 16 h of light. Approximately 50 WBPH (male and female, 2nd and 3rd instar) were inoculated to each group after 10 days of germination, and 3 ppm Kr and Qu were sprayed after each week. The experiment was conducted for five weeks and the survival and death rates of WBPH, development of instars of WBPH, and survival rates of plants and phenotypic evaluation were observed after each week.

### Measurement of water-soluble carbohydrate contents

For quantification of soluble sugar contents, leaves and stem of wild-type and *OxF*_*3*_*H* plants infested with WBPH (discussed in “Test of OxF3H plants’ resistance to WBPH and WBPH rearing”) were collected in three replicates, after 10 days of WBPH infestation, at three time points within one week intervals. Approximately 0.5 g lyophilized tissues were ground into fine powder in liquid nitrogen and homogenized with 80% ethanol (2 ml) at 80 °C for 20 min, following previously described methods^47^. The homogenates were pelleted by centrifugation at 10,000 rpm for 15 min. Supernatants were carefully removed, and the pellets were resuspended in 6 ml distilled water and filtered through 0.2 mm filter paper. Sugar contents were analyzed with HPLC separated with Bio-Rad Aminex 87 C columns (300 × 7.8 mm). Water was used as the eluent at a 0.6 ml/min flow rate.

### Chlorophyll content measurements

Chlorophyll contents of both the wild-type and *OxF*_*3*_*H* plants infested with WBPH (discussed in “Test of OxF3H plants’ resistance to WBPH and WBPH rearing”) were determined using a SPAD-502 chlorophyll meter (Minolta Camera Co., Osaka, Japan), at three time points within one week intervals, following the method described by Lu, et al.^48^. The third leaf of each selected plant was measured after one week of WBPH inoculation. The average of readings from five replicates were taken to measure the mean SPAD measurement for each plot.

### Statistical analysis

All experiments for each section were performed in triplicate, and the data from each replicate were pooled together. Data were analyzed using two-way ANOVA, followed by the Bonferroni post hoc test (significant difference: *p* ˂ 0.05). A completely randomized design was used to compare the mean values of different treatments. Data were graphically presented and the statistical analyses were performed using GraphPad Prism software (version 5.01; GraphPad, San Diego, CA, USA).

## Results

### ***OsF***_***3***_***H*** genetic transformation and generation of the ***OxF***_***3***_***H*** line

The ORF region of the *OsF*_*3*_*H* gene was cloned into a pSB11 expression vector using the gateway system, pENTR Directional TOPO Cloning Kit. Ligation to the pENTR/D-TOPO vector was confirmed by double digestion (Supplemental Figure S1A), while ligation to the expression vectors through LR reaction were confirmed by double digestion and sequencing with company (Supplemental Figure S1B). To prepare the insert, the gene (Os04g0662600, https://www.gramene.org/) was amplified with the forward and reverse primers. The inserted sequence was further evaluated by blasting through the Gramene database (https://www.gramene.org/), and our alignment was 100% identical to the database genome (Supplemental Figure S1C). The gateway system is graphically presented in Supplemental Figure S3, with further details in the Material and Methods section. The *OsF*_*3*_*H* gene was transformed to white calluses using LBA4404 agrobacterium (Supplemental Figure S4A). After two weeks of transformation, green spots appeared on the calluses, which were considered to be the initiation of embryos (Supplemental Figure S4B). The non-gentamycin-resistant calluses turned brown and black, finally dying. After four weeks, embryos emerged from the living callus with gentamycin resistant cells (Supplemental Figure S4C). The embryos carrying the *OsF*_*3*_*H* gene were regenerated into buds, and finally differentiated into leaves in plates (Supplemental Figure S4D). The 18 *OxF*_*3*_*H* plants that were regenerated from the plates were transferred to appropriate medium in test tubes to enhance root growth (Supplemental Figure S4E). After three weeks, the plants were transplanted to soil in a green house and kept until seeds developed (Supplemental Figure S4F). Furthermore the transgenic lines were confirmed by genotyping (Supplemental Figure S5).

### Phenotypic evaluation of rice plants under WBPH stress

**Figure 1:**
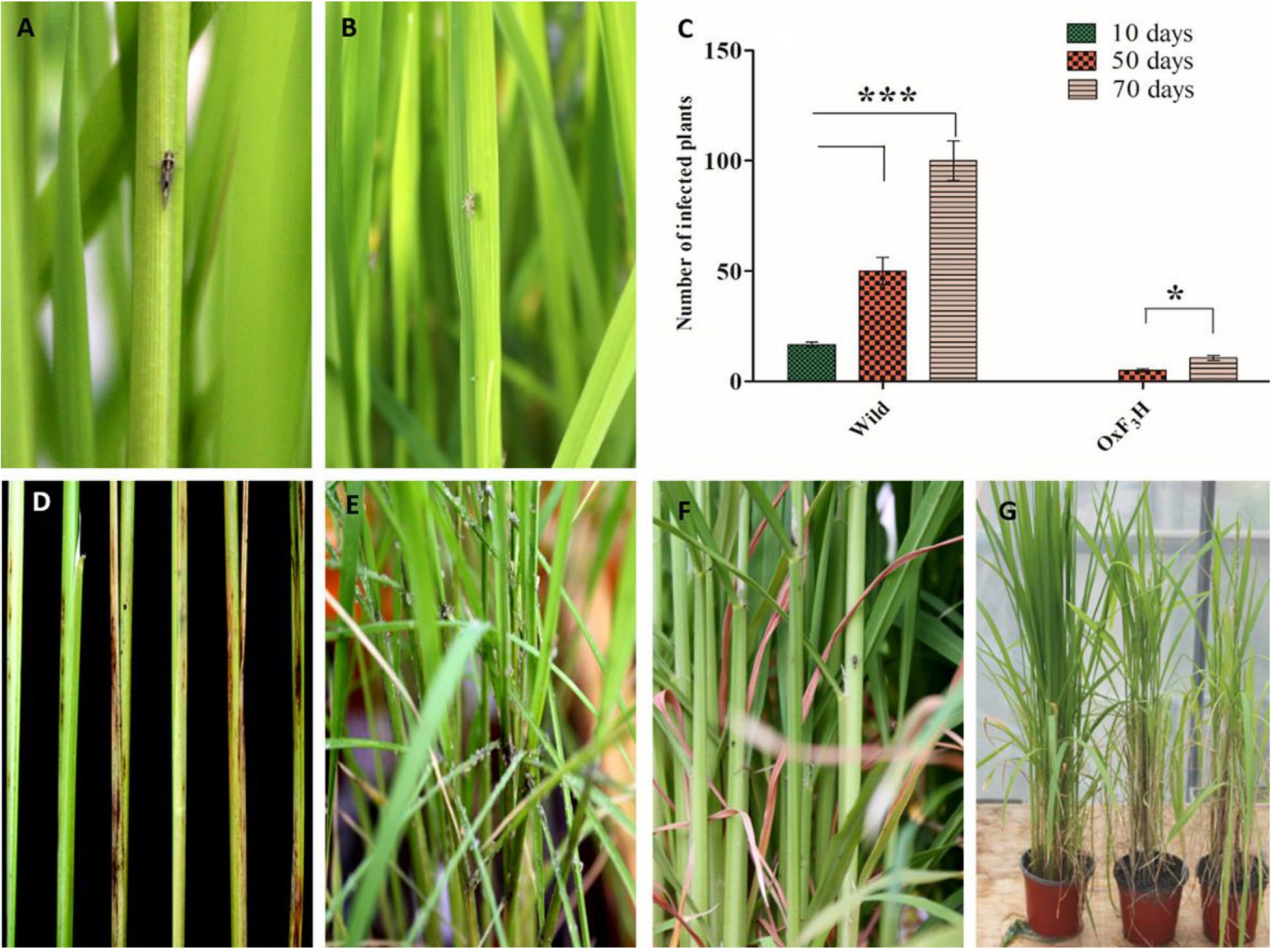
Phenotypic responses of transgenic and wild type rice seedling towards WBPH infestation. (**A**,**B**) represents WBPH attacking site as usually they feeds on stem and leaf midrib due to its sap sucking nature. (**C**) Indicates graphical representation of the number of infected transgenic and wild plants after different time points. (**D**) Lesion length of WBPH infection, the first two stem in left side of the picture shows transgenic plants which shows a minute infection while the rest of four shows wild type infected stem which were badly infected by WBPH, picture taken at later stage of stem development. (**E**,**F**) Wild and transgenic plants respectively which shows the density of WBPH in wild and transgenic line, wild type (**E**) was favorite for WBPH due to huge population present in it and also a huge number of first instar were found in wild type however only a few number of adult WBPH were found in transgenic line (**F**). (**F**) Indicates the effect of continues feeding of WBPH throughout the developmental stages on plant length, first plant of the picture (**G**) at left side indicate transgenic plant while the remaining two plants are wild type which are shorter than transgenic and this feature predicts that due to continuous feeding of WBPH causes dwarfism.

The WBPH susceptible Nagdong cultivar^49^ was used for the *OsF*_*3*_*H* transformation to confirm the presence of phenotypic variations between wild-type and *OxF*_*3*_*H* plants. Phenotypic traits like; shoot length, panicle length, weight of 1,000 grains, and fertility rates of wild-type and *OxF*_*3*_*H* plants were measured (Supplemental Figure S6). The phenotypic variations showed that overexpression of OsF3H also enhances agronomic traits. Such as, shoot length, panicle length, grain weight and fertility rate were higher in transgenic line as compared to wild type. This investigation validated that OsF3H is not only involved in defense mechanism but can also enhance agronomic traits. Previously unrecorded quality data were gathered during phenotypic evaluations under WBPH stress. WBPH are sap-sucking pests that mostly attack stems and leaf midribs (Fig. 1A,B). After WBPH inoculation, the number of infected plants were counted and the infection rate was significantly higher (*p*  ˂  0.05) in wild-type plants. After 10 days of inoculation, 16.6% of the wild-type plants were infected, which increased to 50% and 100%, after 50 and 70 days, respectively. The number of infected *OxF*_*3*_*H* plants was 0%, 5%, and 16.6%, after 10, 50, and 70 days, respectively (Fig. 1C). The lesion length of WBPH infection was also higher in the wild-type plants, visualized at a later stage of development in the stem (Fig. 1D). Considerable color variation was observed for infected wild-type and *OxF*_*3*_*H* plants. Infected wild-type plants were a lighter green than *OxF*_*3*_*H* plants, possibly due to nutrient deficiencies as WBPH are cell sap suckers that can reduced nutrient availability. The feeding ratios of WBPH was much higher in wild-type plants, which was evaluated visually, as quantitative calculations was difficult due to the high population numbers of WBPH. Our results demonstrate that wild-type plants were ideal for WBPH feeding and development, with a large number of first instar larvae found in these plants. Only adult WBPH were found on *OxF*_*3*_*H* plants (Fig. 1E,F, respectively). Only male WBPH were found in *OxF*_*3*_*H* plants, with more female WBPH present on wild-type plants. Our study confirmed that WBPH significantly affects plant length, illustrating that significant WBPH infections with continued feeding causes dwarfism and pre-maturation in wild rice (Fig. 1G).

### Expression of flavonoid-related genes during WBPH stress

**Figure 2:**
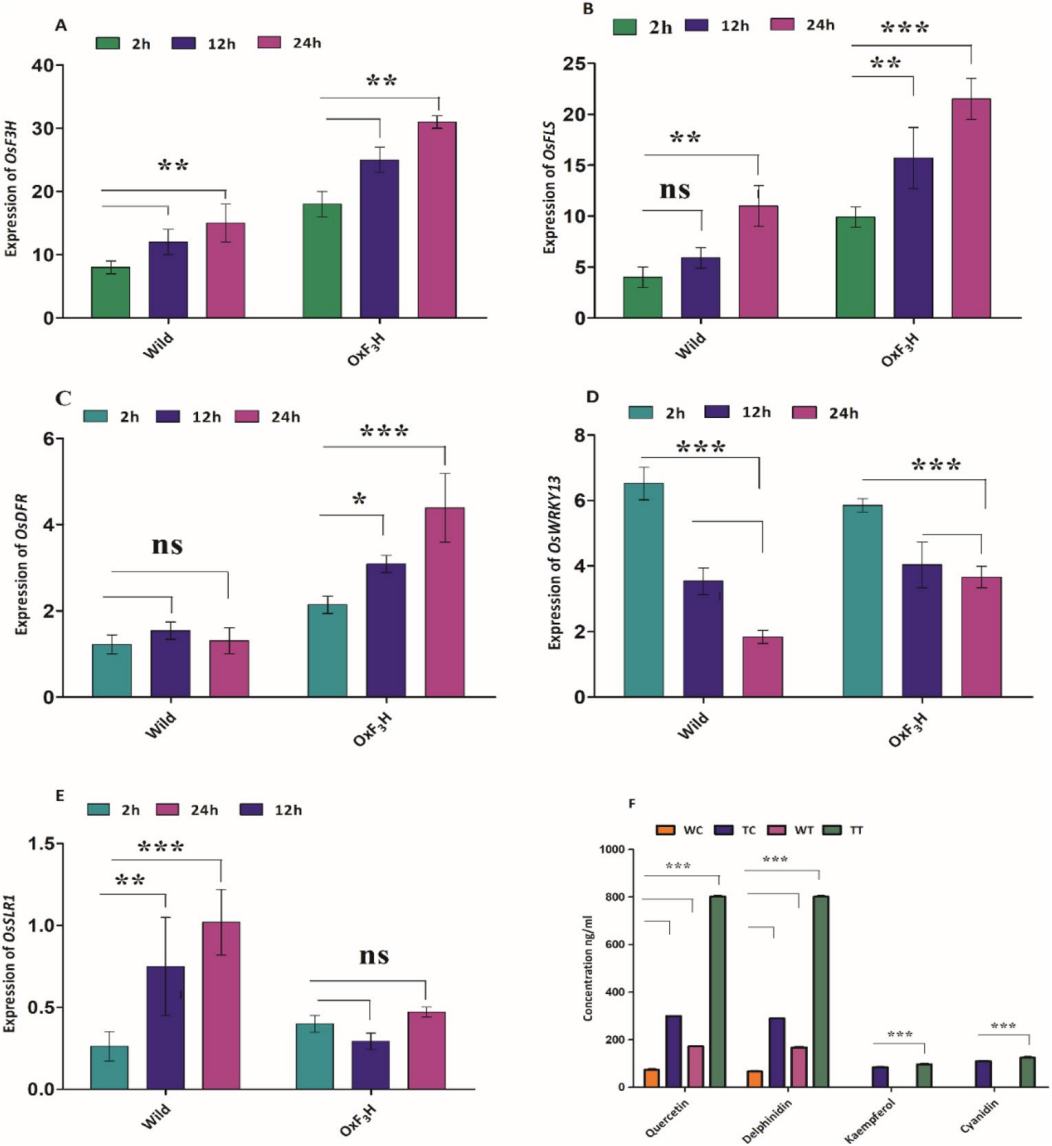
Expression characteristics of *OsF*_*3*_*H* and related genes, and LCMS–MS analysis of flavonol and anthocyanin profiling in response to WBPH stress. Bars represent mean ± standard deviation, asterisks indicate significant difference (*p* ˂ 0.05 two-way ANOVA, Bonferroni post-test) and ns indicate non-significant between wild and transgenic lines. 2 h, 12 h and 24 h represent data taking time points in hours while *OxF*_*3*_*H* indicate transgenic line. (**A**) Expression level of *OsF3H* gene in transgenic line compare to wild type after WBPH infestation. (**B**–**E**) Effect of WBPH induced stress on expression of *OsFLS, OsDFR, OsWRKY13* and *OsSLR1* genes respectively in transgenic plants as compare to wild type. (**F**) LCMS–MS profiling of flavonol (kaempferol, quercetin) and anthocyanin (delphinidin, cyanidin). WC, TC, WT and TT indicate wild control, transgenic control, wild treated and transgenic treated with WBPH respectively. Kaempferol and cyanidin in wild control and wild treated plants.

To evaluate the molecular mechanisms of WBPH-specific induction of flavonol and anthocyanin-related genes, a genetic screening was executed for wild-type and *OxF*_*3*_*H* plants under WBPH stress. Ten leaves from wild-type and *OxF3H* infected plants were randomly selected in triplicate for RNA isolation, to relatively quantify related genes. We investigated the *OsF*_*3*_*H*, *OsFLS*, *OsDFR,* and *OsWRKY13* genes, which are induced by WBPH infestation, using q-PCR analysis (Fig. 2A–D). Results indicated that the patterns of *OsF*_*3*_*H*, *OsFLS,* and *OsDFR* transcription were similar for wild-type and *OxF*_*3*_*H* plants (Fig. 2A–C). However, expression levels were significantly higher (p˂0.05) in *OxF*_*3*_*H* plants, indicating active participation of *OsF*_*3*_*H* in WBPH pest resistance. The *OsF*_*3*_*H* expression levels were 125, 108, and 106 fold higher in *OxF*_*3*_*H* plants than the wild-type after two, 12, and 24 h, respectively. Similarly, *OsFLS* and *OsDFR* expression was enhanced in *OxF*_*3*_*H* plants exposed to WBPH, while *OsFLS* expression was lower in wild-type plants. However, the regulation of *OsDFR* was unclear in wild-type plants, and showed non-significant results at all three time points. These data predict that overexpression of the *OsF*_*3*_*H* gene might lead to the expression of downstream genes related to dihydroflavonols (*OsFLS*) and anthocyanins (*OsDFR*), which facilitate WBPH defense in rice. Flavonols and anthocyanins appear to be involved in the biotic stress response system in rice. The *OsWRKY* transcription factor (Tf) was studied as it facilitates rice resistance by regulating a large number of genes during biotic and abiotic stress^32^. The *WRKY* gene family’s role in pathogen-induced resistance in rice is poorly understood. To evaluate whether *OsWRKY13* facilitates the regulation of genes responsible for signal transduction pathways related to disease resistance, we determined its expression pattern under WBPH induced stress. Expression of *OsWRKY13* was significantly downregulated when comparing wild-type and *OxF*_*3*_*H* plants after WBPH induced stress. Initially, *OsWRKY13* expression in wild-type plants was higher than in *OxF*_*3*_*H.* However, at the second and third time points, expression levels in *OxF*_*3*_*H* plants was increased compared to the wild-type.

### ***OsF***_***3***_***H*** protein expression under WBPH stress

**Figure 3:**
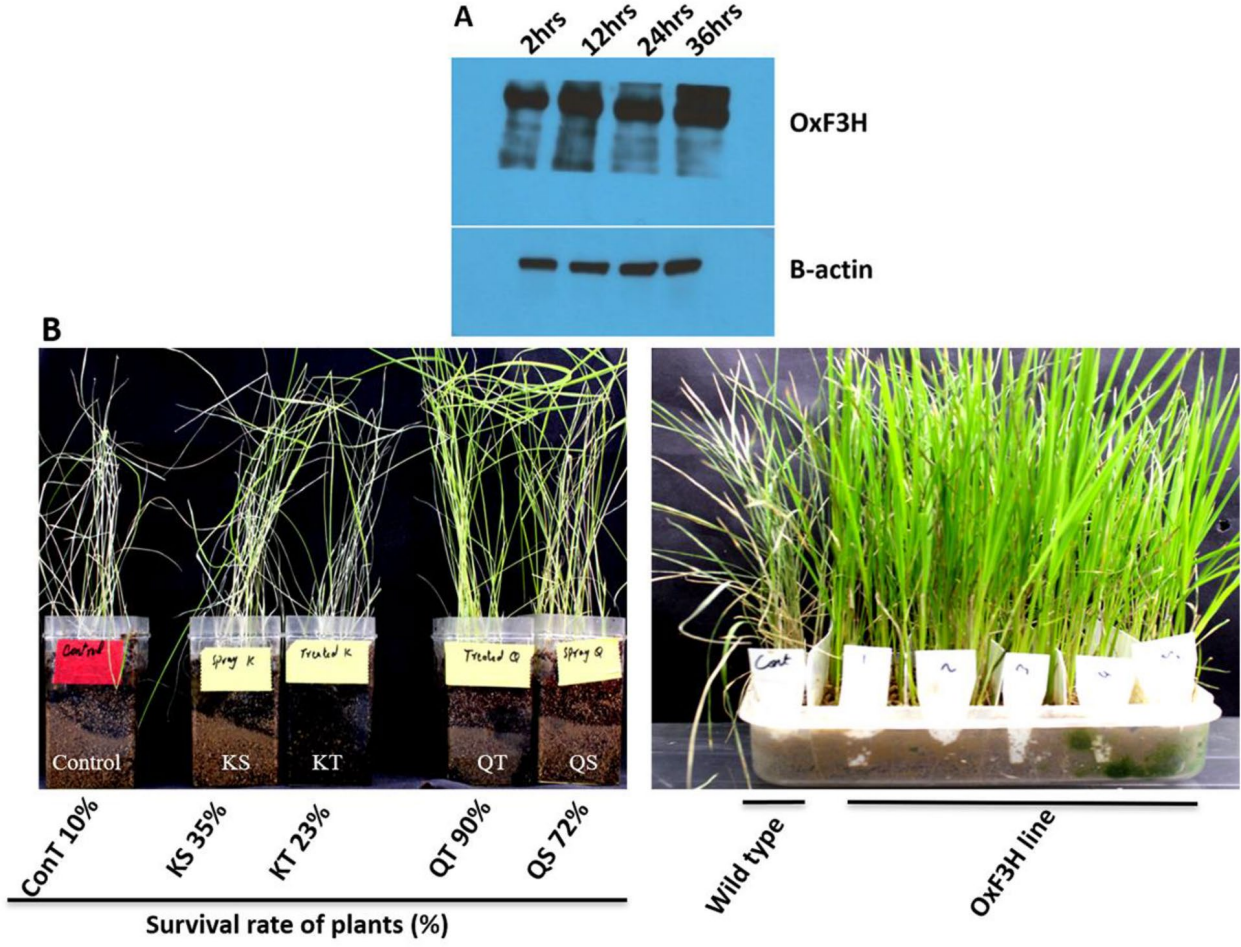
Effect of exogenous application of kaempferol and quercetin on survival rate of plants using susceptible line TN1 and survival difference between wild type and transgenic non-treated plants along with immunoblot analysis of *OsF3H* protein accumulation in transgenic plants. (**A**-upper) Expression of *OsF3H* protein during 2, 12, 24 and 36 h of WBPH infestation to transgenic plants analyzed by western blotting using gene specific antibody. (**A**-lower) β-actin of infected transgenic plants. The β-actin was run with the same sample on the same blot corresponding to the *OsF3H* blot. Figure (**A**) was more cropped because same sample was run in two replicates in separate gels with other samples due to limited time and resources and then transferred to one X-ray film and again replicated on another X-ray film to make it more clear (Figure S8). The blots were cut after the protein transfer to incubate with different antibodies to save time, reagents and materials. (**B**-left picture) KS is kaempferol sprayed, KT is kaempferol treated, QT is quercetin treated and QS is quercetin sprayed. Both the compounds were applied in 3 ppm quantity. The best result shown by quercetin treated and sprayed however, the result of kaempferol treated and sprayed was also better than control plants. (**B**-right picture) Represent the effect of WBPH on susceptible and transgenic line. Control was TNI susceptible to WBPH and the rest represent *OxF3H* line 1–5.

*OsF*_*3*_*H* expression was demonstrated at the protein level through Western blotting in the *OxF*_*3*_*H* line at different time points, during WBPH stress (Fig. 3A). Results showed that WBPH stress positively regulated the *OsF*_*3*_*H* gene. Protein expression was reduced 2 h after WBPH inoculation. However, expression was highest after 12 h and gradually decreased after 24 h, but was still higher than at 2 h. Overexpression of *OsF*_*3*_*H* appears to improve plant resistance to WBPH.

### Kaempferol and quercetin enhanced rice WBPH resistance

Results revealed that *OsF*_*3*_*H* overexpression enhanced WBPH resistance through Kr and Qu induction. Therefore, we further focused our interest to determine the active participation and importance of Kr and Qu, and their roles in regulating WBPH resistance. Initially, we observed the effect of Kr and Qu through exogenous application, using the susceptible TN1 line. Our results showed that the highest plant survival rate of up to 90% was observed in Qu treated plants, and about 72% in Qu sprayed plants. However, comparatively lower plant survival rates of up to 35% and 23% were recorded in Kr sprayed and Kr treated plants, respectively, while control plants showed the lowest (10%) survival rates (Fig. 3B). Phenotypic evaluations also indicated that WBPH adversely damaged control plants, while both Qu and Kr application protected plants against WBPH damage. However, Kr had better results than the control, but lower than Qu (Fig. 3B). Evaluation of WBPH survival rates showed that most WBPH died after the first week of Qu application, followed by Kr, as compared to control plants. The means from 5 weeks of survival rate evaluations showed that WBPH survival rates on control plants was 85%, kaempferol was 37%, and quercetin was 21% (Supplemental Figure S7A,B). Additionally, *OxF*_*3*_*H* plant resistance was checked with susceptible TN1 lines, demonstrating that all TN1 plants died one week after infection. However, *OxF*_*3*_*H* plants exhibited resistance post infection (Fig. 3B). It was also observed that more female than male WBPH died, confirming the increased susceptibility of female WBPH. Large numbers of instars were found in control plants, with fewer in kaempferol-treated plants, and even less in quercetin-treated plants.

### Detection and localization of kaempferol and quercetin in rice seedlings under WBPH stress

**Figure 4:**
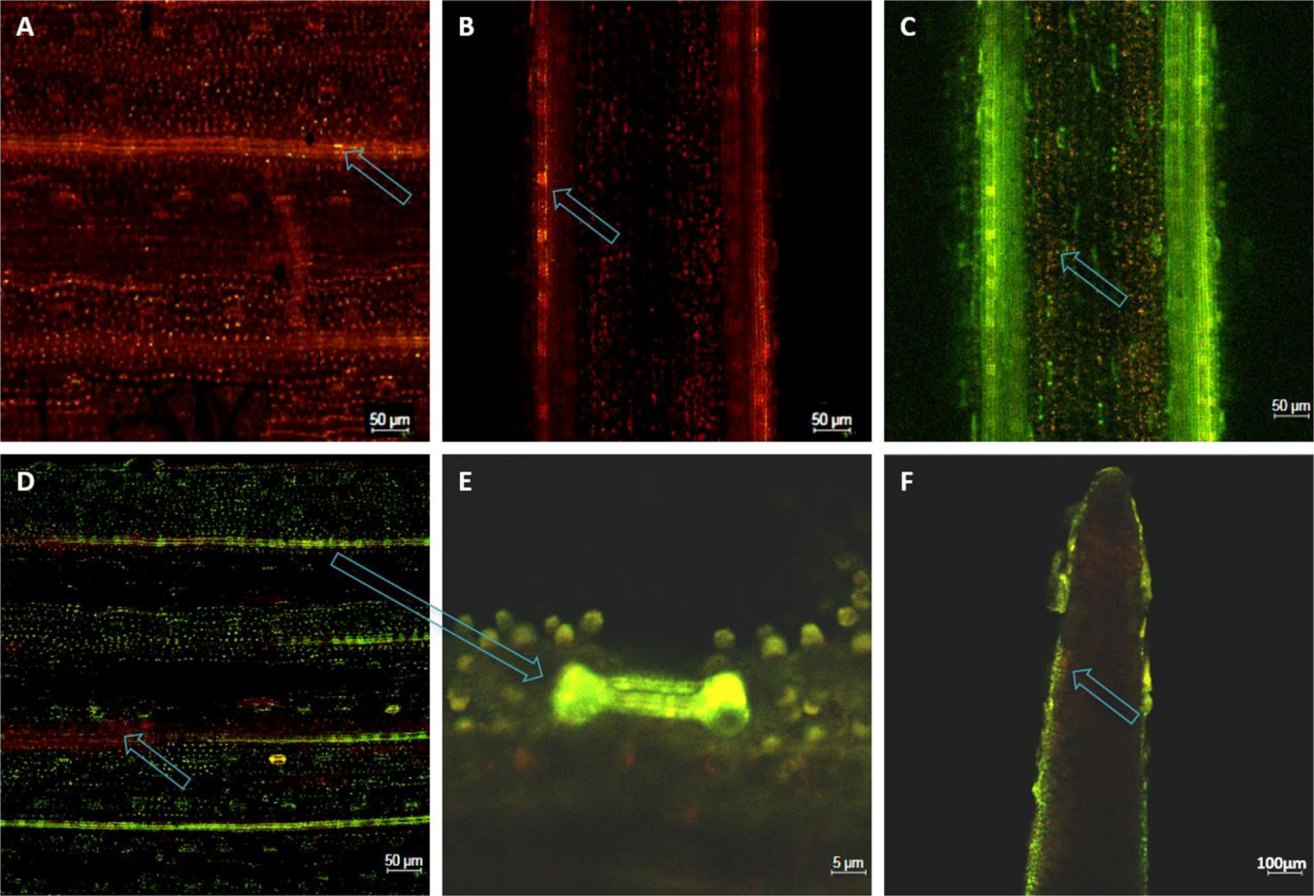
Detection and localization of endogenous kaempferol, quercetin and naringenin in wild and transgenic rice seedling under WBPH stress. All the three compounds were visualized in plant tissue with confocal laser scanning microscope (CLSM) by using florescence dye diphenylboric acid 2-aminoethyl ester (DPBA). Green color indicates kaempferol, orange color indicate quercetin while red color indicates naringenin. (**A**,**B**) Indicate leaf and stem of wild plant having no prominent kaempferol and quercetin and naringenin is dominant however arrows indicates predicted spots of kaempferol and quercetin. (**C**,**D**) Represent stem, leaf and stomata of transgenic seedling respectively. In stem kaempferol was dominant than quercetin in epidermal region however quercetin was dominant in vascular region indicated by arrow. Unlike stem, in leaf quercetin was dominant in stomata region indicated by arrow (**E**) while naringenin was also detected in leaf lamella indicated with arrow (**D**). (**F**) Root tip of transgenic plant indicates both kaempferol and quercetin fluorescence but magnification of kaempferol is higher than quercetin in epidermal tissue while quercetin is higher in interior tissue indicated with arrow.

*OsF*_*3*_*H* functions as part of a regulatory complex of flavonoids that are inhibited by WBPH induced stress. Kr and Qu inhibits stress in vivo, therefore endogenous Kr and Qu accumulation and localization should increase in rice seedlings under WBPH stress. Therefore, we used a flavonoid-specific florescence dye, diphenylboric acid 2-aminoethyl ester (DPBA), for endogenous Kr and Qu differential localization in WBPH stressed rice seedlings. Optical sectioning by confocal laser scanning microscopy (CLSM) exhibited the area of Qu and Kr accumulation, and differentiated Kr and Qu based on differing florescent signals (Fig. 4). Green indicates Kr presence, orange indicates Qu, and red indicates naringenin. We evaluated Kr and Qu presence in the leaves, stems, and roots of wild-type and *OxF*_*3*_*H* plants. Wild-type seedlings exhibited only naringenin accumulation as a red autofluorescence in the leaf and stem area, with an undifferentiated region of Kr and Qu accumulation indicated by arrows in the stomata zone in leaves and the epidermal zone of stems (Fig. 4A,B). This indicates that wild-type plants activate the flavonoid biosynthesis pathway under WBPH stress, but cannot efficiently convert naringenin due to a lack of *OsF*_*3*_*H* activity. However, CLSM analysis of *OxF*_*3*_*H* plants showed that a significant quantity of Kr and Qu accumulated in leaves and stems under stress conditions, with a lower accumulation in roots. This increased leaf and stem accumulation indicates a rapid stress condition response in the form of naringenin conversion, due to *OsF*_*3*_*H* overexpression. Kr mostly accumulated in the epidermal regions and stomata cells, while Qu accumulated in the xylem and phloem (Fig. 4C). Accumulation was also higher in *OxF*_*3*_*H* plant leaves, with most of the Kr localized to stomata cells and some detected in mesophyll cells. A small quantity of naringenin was observed in leaf mesophyll tissue (Fig. 4D). Due to high Kr accumulation in stomata cells, it was predicted that rice plants close the stomata under WBPH induced stress (Fig. 4E). Kr and Qu were also detected in roots, with higher Kr levels compared to Qu, indicated by the arrow. Kr localized to the epidermal regions, while Qu was present in interior cells (Fig. 4E).

### Comparison of flavonols and anthocyanin in response to WBPH

We also determined the flavonol (kaempferol, quercetin) and anthocyanin (delphinidin, cyanidin) concentrations in relation to the flavonoid biosynthesis pathway (Fig. 2F). WBPH treated plants exhibited remarkable variation in flavonol and anthocyanin accumulation, compared to non-treated plants. We found that Qu and delphinidin accumulation was increased in *OxF*_*3*_*H* treated plants (802.7, 801.6 ng/g respectively), followed by *OxF*_*3*_*H* control plants (299.5, 289.8 ng/g respectively), indicating that *OsF*_*3*_*H* overexpression significantly (p < 0.05) induces flavonol production and anthocyanins biosynthesis. Kr and cyanidin were induced in *OxF*_*3*_*H* treated and -control plants, but with much lower accumulation levels compared to that of Qu and delphinidin. Qu and delphinidin were also produced in low quantities in wild-type treated and -control plants, but Kr and Qu were not detected in either the treated or controlled plants. The standard equations and a table of validation methods for related compounds are shown in Supplemental Tables S3 and S4 respectively.

### ***OsF***_***3***_***H*** gene regulates JA biosynthesis

**Figure 5:**
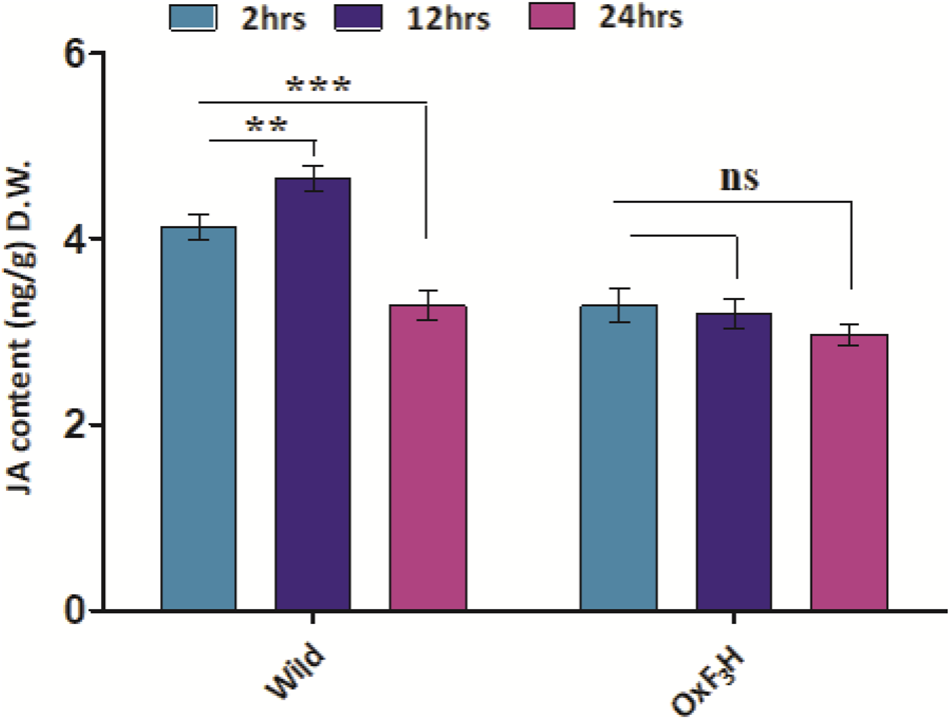
JA regulation in wild and transgenic plants during WBPH stress after 2, 12 and 24 h.

We quantified the JA in wild-type and *OxF*_*3*_*H* plants to evaluate whether *OsF*_*3*_*H* overexpression induces JA under biotic stress. After exposing both wild-type and *OxF*_*3*_*H* plants to WBPH, JA levels increased in the wild-type plants at each time point, but were not significantly affected in the *OxF*_*3*_*H* plants. The amount of JA in wild-type plants increased by 20% after 2 h, 31% after 12 h, and 9% after 24 h, in comparison with *OxF*_*3*_*H* plants (Fig. 5).

### ***OsF***_***3***_***H*** overexpression suppresses ***OsSLR1*** to mitigate dwarfism

*SLR1* is one of the key genes that encodes the DELLA protein and functions as a GA response suppressor in the GA signaling pathway. Our phenotypic study found that high WBPH numbers caused devastating damage to rice seedlings and plant death (Fig. 3B). However, a large population of WBPH in the developing stage caused severe damage, dwarfism, and pre-maturation (Fig. 1G). To determine the mechanism of how *OsSLR1* causes dwarfism and pre-maturation at a transcriptional level under WBPH induction, we identified the *OsSLR1* expression levels at different time points in wild-type and *OxF*_*3*_*H* plants. *OsSLR1* was significantly (*p* < 0.05) upregulated in wild-type plants, with irregular expression in *OxF*_*3*_*H* plants (Fig. 2E). In *OxF*_*3*_*H* plants, *OsSLR1* was upregulated 2 h after infestation, downregulated at the second timepoint, and upregulated again at the third time point. Thus, *OsSLR1* expression seems to influence dwarfism and pre-maturation of wild-type plants.

### Quantification of sugar and chlorophyll contents under WBPH infestation

**Figure 6:**
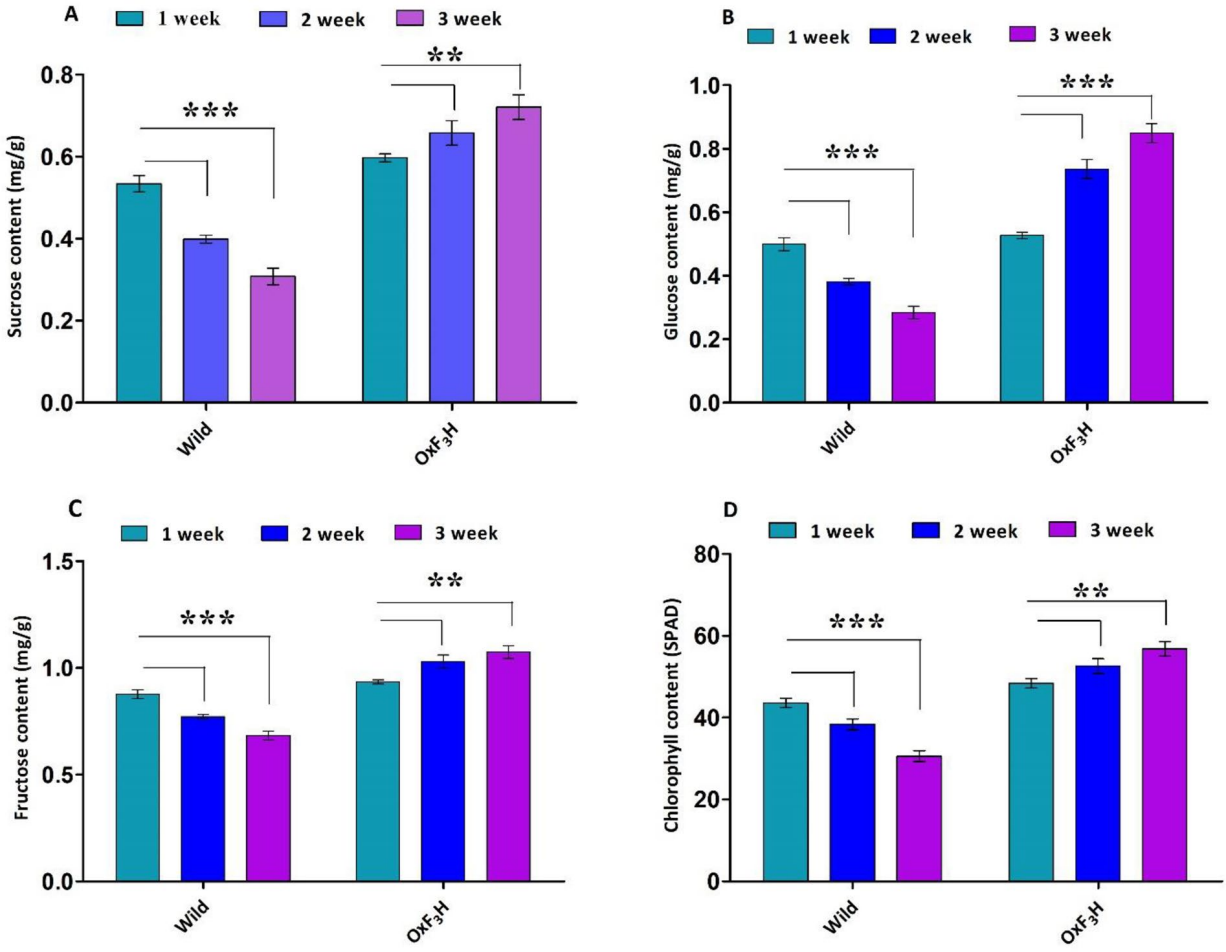
Sugar contents, chlorophyll contents and JA quantification. Bars represent mean ± standard deviation, asterisks indicate significant difference (*p* ˂ 0.05 two-way ANOVA, Bonferroni post-test) and ns indicate non-significant between wild and transgenic lines. 1 week, 2 week and 3 weeks represent data taking time points in transgenic as well as wild type plants. (**A**–**C**) Sucrose, glucose and fructose quantification respectively under WBPH stress. (**D**) Chlorophyll contents accumulated after each week of WBPH infestation by SPAD.

WBPH infestations significantly reduced sugar content (Fig. 6B–D). Analyses showed decreased amounts of sucrose, glucose, and fructose in wild-type plants at each time point, while *OxF*_*3*_*H* plants showed significant increases (*p* < 0.05). In wild-type plants, sucrose, glucose, and fructose were decreased by 42%, 43%, and 22%, respectively, between the first and last time points, and increased by 20%, 61%, and 14%, respectively, in *OxF*_*3*_*H* plants. Chlorophyll content was also reduced by 29% in wild-type plants and increased by 17% in *OxF*_*3*_*H* plants, between the first and last time points (Fig. 6D).

## Discussion

We selected an appropriate gene, *OsF*_*3*_*H*, by using quantitative trait locus (QTL) data, after WBPH rice plant infestation. The WBPH response in the *OxF*_*3*_*H* F1 generation was evaluated significant differences were noted between transgenic and wild plants (Fig. 1C-G). Our results indicated that WBPH prefer wild-type plants, confirmed by the presence of larger feeding populations and the increased number of infected wild-type plants (Fig. 1C). The preference of WBPH for wild-type rice could be due to the high flavonoid biosynthesis rates in *OxF*_*3*_*H* plants. Our results also identified that wild-type lesion lengths were higher than in *OxF*_*3*_*H* (Fig. 1D), due to high lignin biosynthesis levels that increases the cell wall mechanical support, possibly reducing tissue damage. Previous reports have shown that oxidative stress activates the phenylpropanoid metabolic pathway related genes involved in the synthesis of lignin and flavonoids^50^. It is possible that *OsF*_*3*_*H* overexpression could produce more lignin due to its prominent position in the flavonoid biosynthesis pathway, potentially providing a mechanical defense mechanism against WBPH infestation.

**Figure 7:**
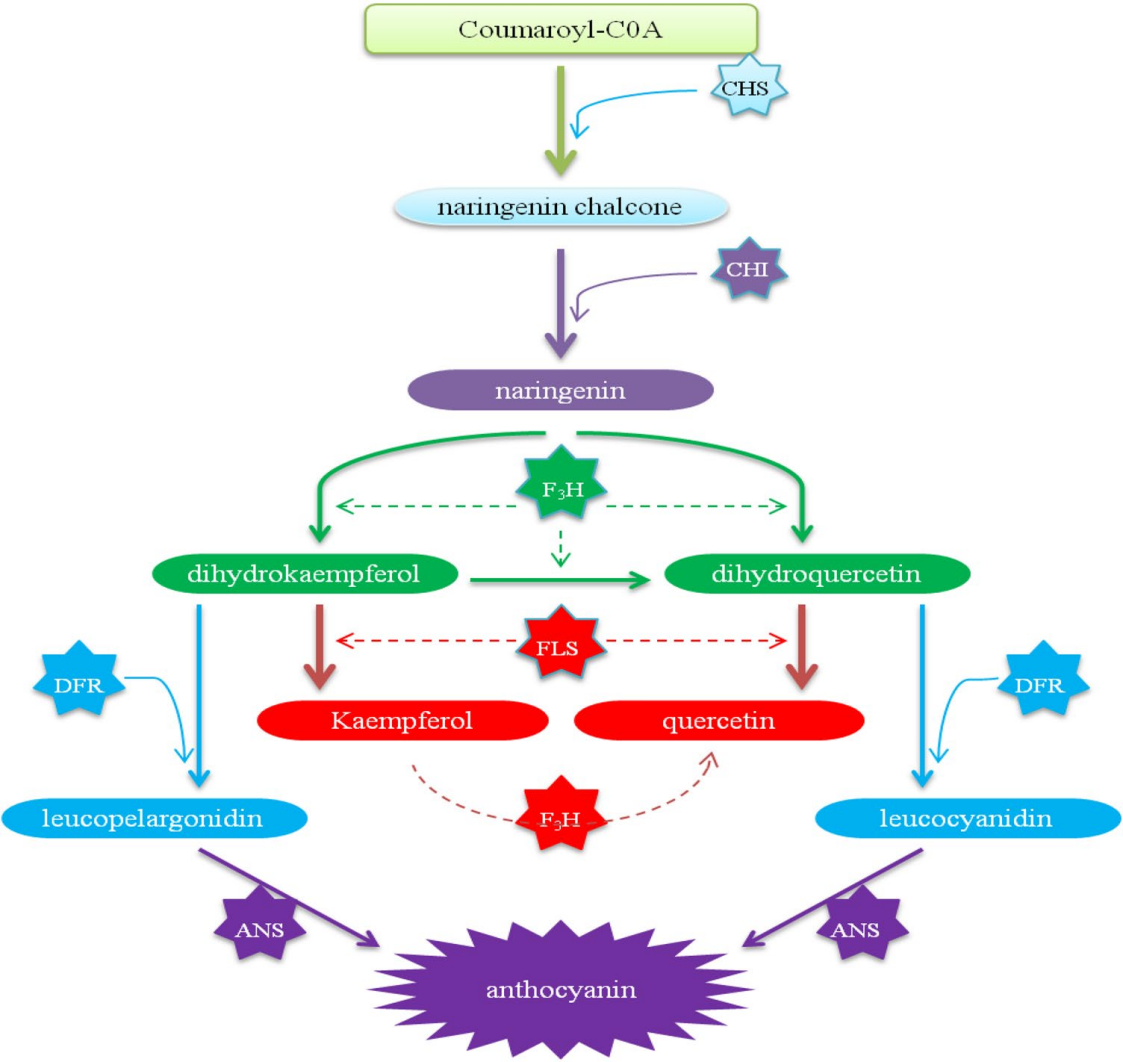
Schematic representation of molecular regulatory network of kaempferol, quercetin and anthocyanin biosynthesis in flavonoid pathway in rice. Enzymes involved in each step: *CHS* chalcone synthase, *CHI* chalcone isomerase, *F3H* flavanone 3-hydroxylase, *FLS* flavonol synthase, *DFR* dihydroflavonol reductase, *ANS* anthocyanidin synthase.

*OsF*_*3*_*H* is a prominent gene in the flavonoid biosynthesis pathway, supporting the association of flavonoids with WBPH resistance. To confirm whether *OsF*_*3*_*H* induces WBPH resistance and the regulation of downstream genes, we carried out a genetic functional analysis through overexpression. Our results indicated that WBPH regulates the *OsF*_*3*_*H* gene in wild-type and *OxF*_*3*_*H* rice, but that transcriptional and translational expression was significantly higher in *OxF*_*3*_*H* plants (Figs. 2A, 3D). This shows that *OsF*_*3*_*H* actively participated in WBPH resistance, and positively regulated WBPH response characteristics and flavonoid accumulation. Furthermore, in addition to *OsF*_*3*_*H*, our study extends to the regulation of the downstream signaling genes, *OsFLS* and *OsDFR*, during WBPH infestation. These genes are involved in flavonol and anthocyanin synthesis. *OsF*_*3*_*H*, *OsFLS,* and *OsDFR* were also significantly upregulated in *OxF*_*3*_*H* plants during WBPH infestation. A previous report showed that the activity of downstream genes responsible for the reduction of flavonol and anthocyanin accumulation were reduced in rice tissues, due to a lack of *F*_*3*_*H* gene activity^51^. Researchers evaluated the function of *OsF*_*3*_*H* in flavonoid biosynthesis and BPH resistance, through the development of *OsF*_*3*_*H* overexpression and the use of RNAi in plants. Results showed that overexpressing plants were more resistant, and plants treated with RNAi were more susceptible, compared with wild-type plants. Additionally, flavonoid contents were increased in overexpressing-, and decreased in RNAi plants^52^. *OsF*_*3*_*H*, *OsFLS*, and *OsDFR* genes were co-regulated as one regulatory unit during WBPH induced stress, indicating that flavonoid, flavonols, and anthocyanins are synthesized as a single unit during stress conditions. Our results predict that *OsFLS,* which is not a structural gene, is co-expressed *OsF*_*3*_*H*. The *OsF*_*3*_*H* gene converts naringenin into dihydrokaempferol or dihydroquercetin (flavanonol), which is used by *OsFLS* as a substrate to synthesize Kr and Qu (flavonol) (Fig. 7)^52^. The TF families of *WRKY* and *MYB* are broadly involved in regulating various metabolic pathways in plants under stress conditions^57^. To demonstrate whether the *OsWRKY13* TF significantly regulated genes involved in other signal transduction pathways responsible for disease resistance, we analyzed its quantitative expression during WBPH stress in rice plants. We found that *OsWRKY13* expression was initially higher, but was downregulated at later stages of WBPH infestation. However, expression was still higher in the wild-type than in the *OxF*_*3*_*H* plants. *OsWRKY13* TF downregulation could be due to *OsF*_*3*_*H* overexpression, which can enhance the production of downstream genes responsible for the biosynthesis of flavonols and anthocyanins, which are responsible for stress resistance. We expected a downregulation of *OsWRKY13* in wild-type plants after 12 and 24 h of WBPH infestation, due to the infection severity caused by the high densities and extended feeding times of WBPH. However, *OsWRKY13* expression was higher in the initial stages. Reports show that the *WRKY* TF binding site (a W-box) occurs in the *F*_*3*_*H* and *DFR* promoter regions, regulating various processes related to defense against pathogenic and abiotic stressors^57^. Previous investigation additionally reported that *OsWRKY13* induces the *F*_*3*_*H* gene^31^. This observation provides strong evidence that *WRKY* TF is involved in plant resistance against WBPH induced stress, via the induction of gene expression in the flavonoid biosynthesis pathway.

We further extended our experiments to include the exogenous application of Kr and Qu against WBPH (Fig. 3B). We found that Kr and Qu have significant pesticidal properties against WBPH, with Qu having a greater effect than Kr (Fig. 3B). Visual observations showed that the WBPH death rate was higher with exogenously applied Kr and Qu, compared to untreated plants. This indicates that Kr and Qu are strong pesticides which adversely inhibit insect digestion, acting as deterrents of WBPH. A previous investigation reported that rice flavone glucosides are strong inhibitors of *Nilaparvatalugens* digestion, acting as a deterrent^58^. Qu glucoside also increases mortality and inhibits *Lymantria dispar* and *Spodopteralitura* development^59^. Kr and Qu also have repellent properties against nematodes, which feed on plants like *Radopholussimilis* and *Meloidogyne incognita*^60^. Our observations indicated that the development rates of newly hatched nymphs was negligible, supporting the assertion that Kr and Qu inhibits hatching. Kr restricts hatching, and some other flavonoids prevent insects from laying eggs^61,62^. Kr and Qu are strong ROS scavengers that reduce oxidative stress in cells^63,64^. Confocal microscopy provided strong evidence for Kr and Qu accumulation in various seedling tissues under stress conditions, when stained with DPBA (Fig. 4). Our results showed that Kr and Qu accumulated in rice seedlings, parallel with *OsF*_*3*_*H* and *OsFLS* expression under WBPH induced stress, as previously reported^66^. Naringenin accumulated at high levels in leaves and stems of non-infected plants, indicating that it is not converted into Kr and Qu under normal conditions (Fig. 4A,B). However, large amounts of Kr and Qu accumulated in the epidermal region of stems and the stomata of leaves in infected plants (Fig. 4C,D). A smaller amount of naringenin was detected in leaves (Fig. 4D). Large amounts of observed Kr in the epidermal region of stems suggests that it initially accumulates in the infectious region to protect the plant from damage. Kr and Qu are released in vascular tissues for transportation to different parts of the plant to boost defensive mechanisms against the pathogen throughout the entire plant. This observation indicates that naringenin is an unstable compound and is rapidly converted into Kr and Qu when exposed to stressors. A previous report indicated that the flavonoid biosynthetic pathway is under feedback control, with naringenin inducing the transcription of genes encoding its biosynthetic enzymes^66^. This result supports the hypothesis that naringenin is rapidly converted due to WBPH induced gene expression. The presence of naringenin in the stem vascular regions indicates that conversion is enhanced only in epidermal tissues due to direct contact with WBPH, as naringenin was only detected in vascular regions (Fig. 4C). Kr and Qu were also highly localized to the stomata, possibly keeping the stomata closed during WBPH infestation. Stomata closure is a defensive mechanism against stressors. However, low accumulation in the roots of infected plants indicates a reduced response to WBPH induced stress.

Flavonols and anthocyanins have received attention as indicators of pest and pathogen resistance, and excessive light, drought, and cold stress^67^. Although the role of flavonols and anthocyanin against abiotic stressors in plants is well documented, their role in plant–herbivore interactions remains unclear^68^. Here, we observed that Kr, Qu, delphinidin, and cyanidin levels in the *OxF*_*3*_*H* treated line were significantly (*p* < 0.001) higher than in *OxF*_*3*_*H* non-treated and wild-type treated plants (Fig. 6A). The levels of related compounds in the *OxF*_*3*_*H* non-treated line was also higher than in the wild-type control and treated plants. High flavonol and anthocyanin levels provides strong evidence of *OxF*_*3*_*H* line resistance against WBPH induced stress. Our results showed that Kr and cyanidin did not accumulate in wild-type plants, indicating notably low production not detected by LCMS–MS, or the possibility that these are not regulated during WBPH induced stress. Similar results were observed in confocal laser microscopy analysis where Kr was not detected in wild-type plants, while Qu was detected at low magnification. (Fig. 4A,B). Previous reports suggested that Kr constitutes lower antioxidant activity compared to Qu, which is a strong antioxidant, and that Qu biosynthesis was higher than Kr during the stressed condition^70^, which favors our result of lower Kr production compared to Qu. It has been reported that *MYB75* overexpression*,* which is involved in the flavonoid biosynthesis pathway, significantly regulates anthocyanins, Kr, and Qu biosynthesis during caterpillar or aphid stress, with increased levels in over-expressors compared to wild-type plants. Kr and Qu have been shown to accumulate in cotton crops during insect feeding, while anthocyanins were effective in bacterial blight resistance in cotton leaves^71^. Results from LCMS–MS and the peaks detected for different compounds are presented in Supplemental Figure S8.

Additionally, high-density WBPH infestation caused rice seedling death, but led to pre-maturation and dwarfism, and eventually decreased yield in the tillering stage. Previous researchers reported that WBPH infestation significantly reduced shoot length and plant vigor, enhanced leaf discoloration, and inhibited tillering emergence and grain setting^72–74^. These results also support our finding regarding the decreased chlorophyll contents of wild-type plants, and its regulation in *OxF*_*3*_*H* plants under stress conditions (Fig. 6D). To investigate the cause of dwarfism, we extended our study to *OsSLR1*, which produces the DELLA protein, enhances immunity, and regulates the JA hormone during WBPH induced stress. We found that *OsSRL1* significantly enhanced defenses against WBPH in wild-type plants due to lack of *OsF*_*3*_*H* activity. However, due to *OsF*_*3*_*H* expression in *OxF*_*3*_*H* plants, *OsSLR1* expression was reduced, although non-significantly. This suggests that *OsSLR1* enhances the basal defensive mechanism under stressed conditions. Figure 1E shows that high-density infestation inhibited growth in wild-type plants, although the plants still survived possibly due to the activation of plant defensive mechanisms, which usually take place at the expense of growth. Previous reports investigated whether this conflict between growth and defense is supported by the principle that plant-resources are restricted and can only be utilized for either growth or defense, depending on exterior and interior conditions^75^. In *OxF*_*3*_*H* plants, stress was reduced due to *OsF*_*3*_*H* overexpression, resulting in the inhibition of *OsSLR1* expression. Researchers found that the *SLR1* gene positively regulates the defense mechanism by regulating the SA and JA signaling pathways^76^. Although we did not evaluate GA regulation, previous investigations have reported antagonism of JA and GA. Our results identified positive regulation of JA in wild-type plants, compared to *OxF*_*3*_*H* plants, providing strong evidence for GA restriction under stressed conditions, resulting in growth inhibition^75,77^. However, decreased JA levels in *OxF*_*3*_*H* plants avoids stress mitigation induced by WBPH, through *OsF*_*3*_*H* expression. The mutual antagonism between JA-GA is an important strategy in maintaining the balance between defense and growth, through physical interactions between DELLAs and JAZs^75,77^. These results indicate that along with *OsF*_*3*_*H*, DELLA proteins also contribute to defense against WBPH stress, whether in the form of JA upregulation—which is the ultimate source of anthocyanin biosynthesis, or in the form of hijacking of the GA crosstalk mechanism.

Along with the induction of the *OsSLR1* gene and JA-GA antagonistic mutualism, sugar content reduction is another inhibitor of plant length. Sugar is crucial for plant growth and development as it is a major source of energy. It has been reported that exogenous application of sugar enhanced plant growth. Sugar also upregulates genes associated with the defense mechanism and biosynthesis of secondary metabolites. Sugar content inhibition in wild-type plants is also associated with slow photosynthesis rates due to a decrease in chlorophyll contents and the high density of WBPH feeding which extract sugar and other nutrients from the phloem. The second hypothesis for the decrease in sugar content in wild-type plants is the deviation of total energy toward defense mechanisms, which utilizes large quantities of sugar as a carbon source. The use of large quantities of sugar as a defensive tool critically affects plant growth and development, ultimately resulting in stunted growth.

## Conclusion

WBPH is a significant biotic stressor that can seriously impact rice yield in several countries. Previously, conventional breeding was the main tool for selecting the most effective, easily adaptable, and resistant crop varieties. However, molecular breeding techniques are currently used to protect agricultural crops by developing new, resistant variants. Using molecular breeding techniques, we developed a highly WBPH-resistant *OxF*_*3*_*H* rice cultivar by selecting the gene of interest through QTL analysis. Plants respond to pest attacks through a complex network of transcriptional, proteomic, metabolomic, and phytohormonal reprogramming. In this study, we evaluated all possible mechanisms of regulation of complex responses, like regulation of the *OsF*_*3*_*H* gene at RNA, protein, metabolite, and hormone levels. *OsF*_*3*_*H* overexpression in rice, which resulted in elevated anthocyanins and flavonol production, was used to study the potential roles of anthocyanins and flavonols in plant–WBPH interactions. Overexpression of *OsF3H* has been reported to upregulate structural genes of the flavonoid biosynthesis pathway and its related proteins in *OxF3H* plants, while directly or indirectly downregulating JA. The results also demonstrated that the expression of structural anthocyanins and flavonols biosynthesis pathway genes in *OxF3H* plants were higher than in wild-type plants, demonstrating that anthocyanins and flavonols are crucial to the WBPH response. This is novel information identifying that anthocyanins and flavonols, especially Qu, plays a key role in WBPH resistance. Similarly, our study confirmed Qu as a significant deterrent of WBPH when applied exogenously, which predicts that Qu could be used as strong pesticide. Phenotypic evaluations of *OxF3H* plants, compared to wild-type plants, provided significant evidence that anthocyanins and flavonols play a critical part in producing WBPH resistance.

## Supplementary information

Supplementary file 1
