# Supplementary material for: Overexpression of OsF3H modulates WBPH stress by alteration of phenylpropanoid pathway at a transcriptomic and metabolomic level in Oryza sativa
Source: Sci Rep. 2020 Sep 7;10:14685. doi: 10.1038/s41598-020-71661-z (PMC7477192; doi:10.1038/s41598-020-71661-z)
Supplement: Supplementary file 1 [file 41598_2020_71661_MOESM1_ESM.docx]

**Overexpression of *OsF_3_H* modulates WBPH stress by alteration of phenylpropanoid pathway at transcriptomic and metabolomic level in *Oryza sativa***

Rahmatullah Jan^1^, Muhammad Aqil Khan^1^, Sajjad Asaf^2^, In-Jung Lee^1^, Kyung-Min Kim^1*^

^1^Division of Plant Biosciences, School of Applied Biosciences, College of Agriculture & Life Science, Kyungpook National University, 80 Dahak-ro, Buk-gu, Daegu, 41566, Republic of Korea

^2^Natural and Medical Science Research Center, University of Nizwa 616, Oman

^*^Corresponded authors; K.M. Kim, [kkm@knu.ac.kr](mailto:kkm@knu.ac.kr)

Phone # +82-53-958-6880


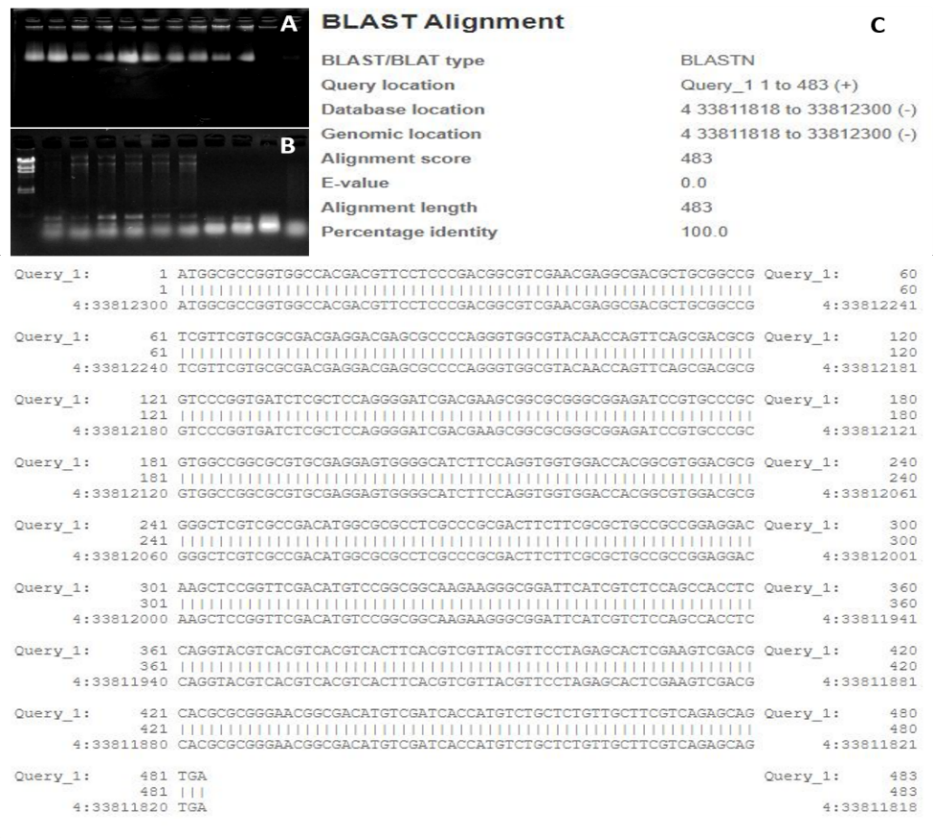


**Figure S1.** Confirmation of ligation reaction through gel electrophoresis. (A) Confirmation of entry vector through double digestion with Not1 and Asc1 enzyme. (B) Double digestion of binary vector with BamH1 and Xho1 enzyme. (C) Sequence query of *OsF3H*.


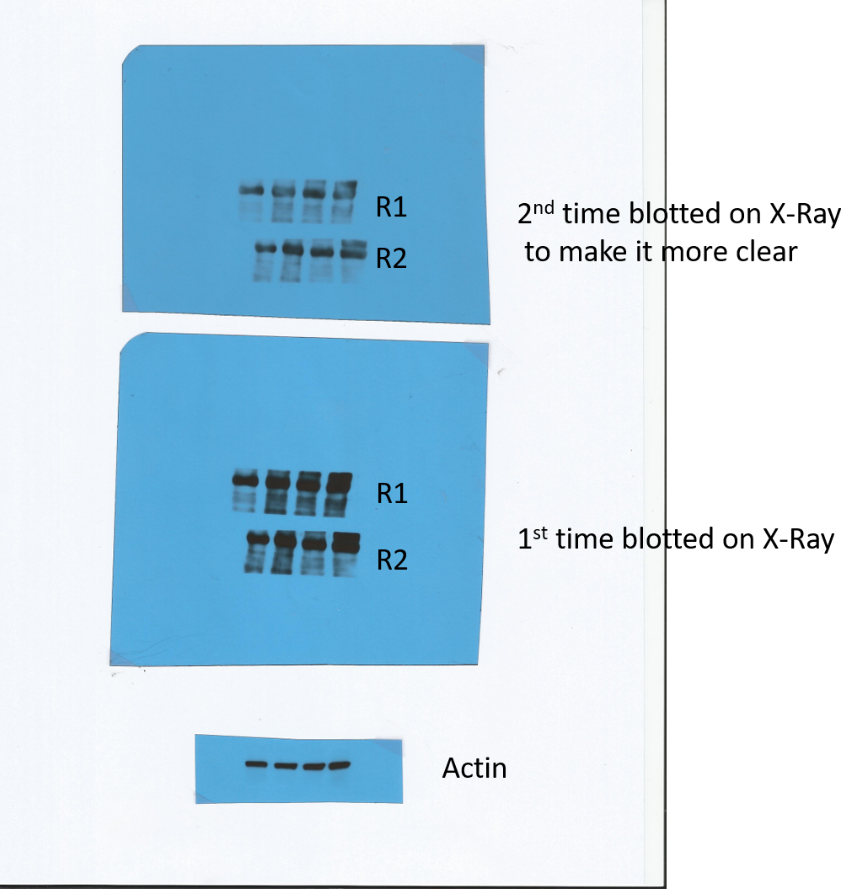


**Figure S2.** This is figure shows two replicates of same samples run on two different gel. R1 is the first gel and R2 is the second gel while 1^st^ and 2^nd^ time blotted figures are just the replication to more clear the bands. These gels were blotted together with the other samples on one X-ray film and then we cropped accordingly and collected the X-ray pieces in one picture.


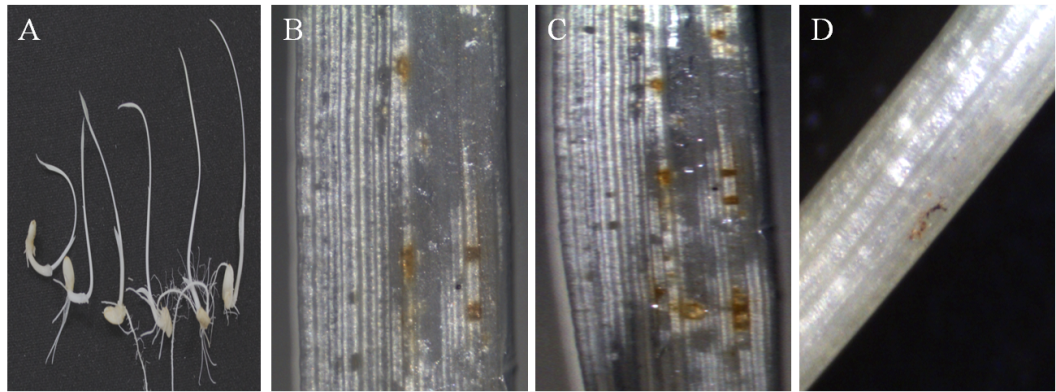


**Figure S3.** Development of albino seedling grown on 3ppm norflurazon herbicide. (A) Represent albino seedling, (B and C) indicate WBPH symptoms on leaves and (D) represent symptom on stem.


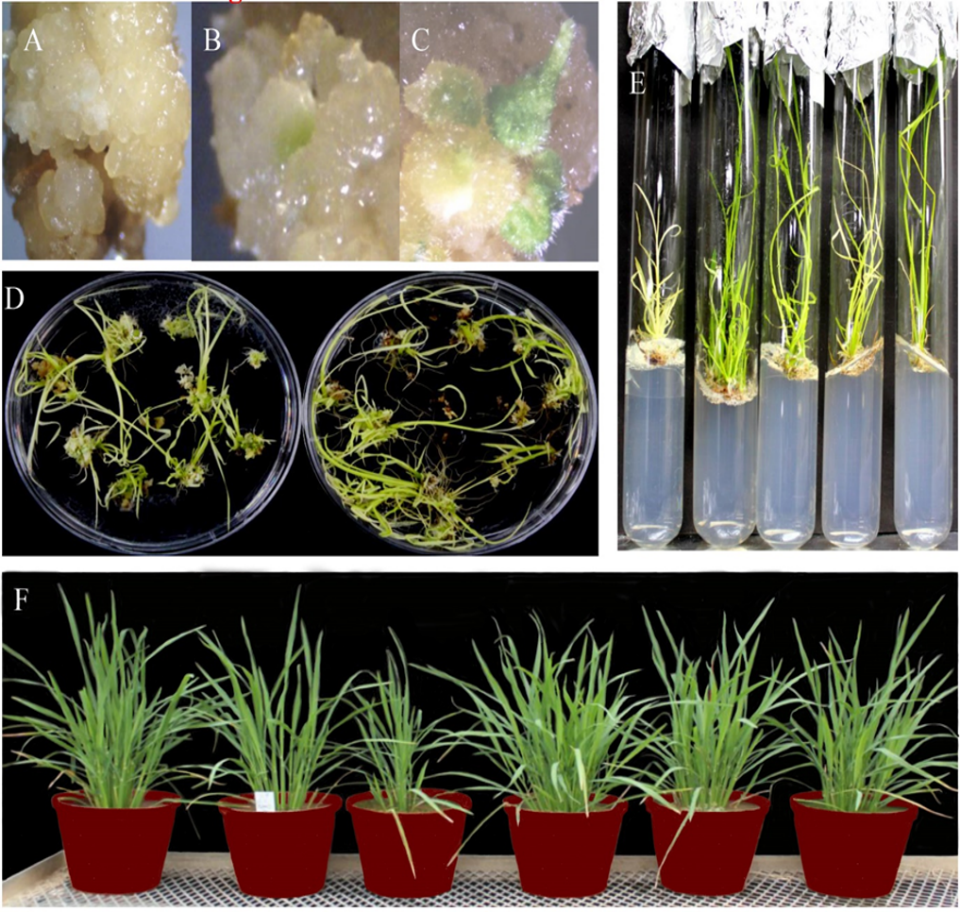


**Figure S4.** Graphical representation of development of transgenic plants via tissue culture technique. (A-C) Morphological changes and development of green spot after agrobacterium transformation on selection media; (D and E) development of shoot and root in regeneration medium; (F) development of complete transgenic plant in soil in green house.





**Figure S5.** Genotyping of wild and overexpressor line. The first 6 bands of F3H gene was amplified with gene forward and revers primers using DNA of wild type and overexpressor line respectively. While the last three bands were amplified from the DNA extracted from overexpressor line using 35S promoter forward and gene revers primer. The empty slot shows that the gene was not amplified while using wild type DNA and 35S promoter forward and gene revers primer.


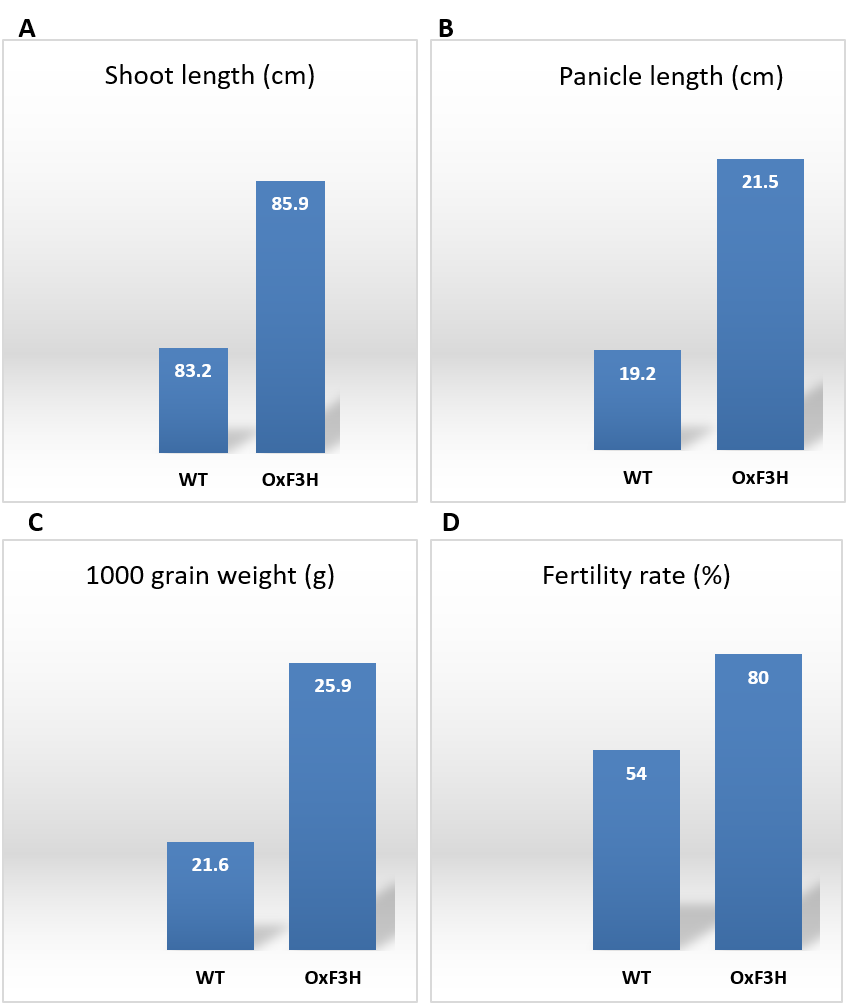


**Figure S6.** Phenotypic evaluation of wild and OxF3H plants. (A) shoot length, (B) panicle length, (C) weight of 1000 gains and (D) fertility rate.


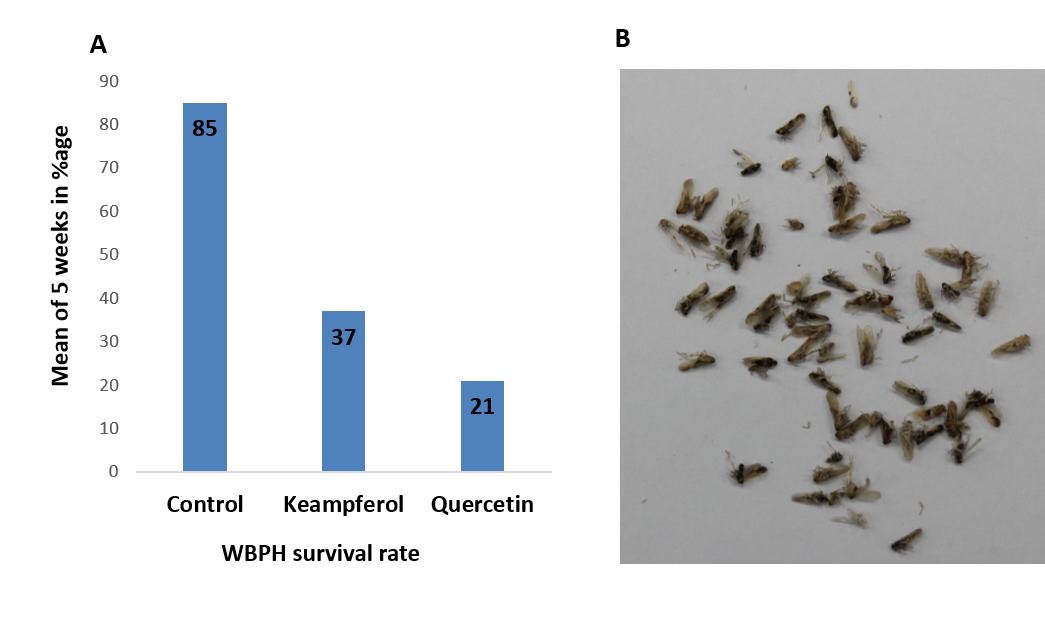


**Figure S7.** Survival rate of WBPH on kaempferol and quercetin treated plants. (A) percentage of WBPH survived and (B) the image of dead WBPH due to kaempferol and quercetin treatment.

**
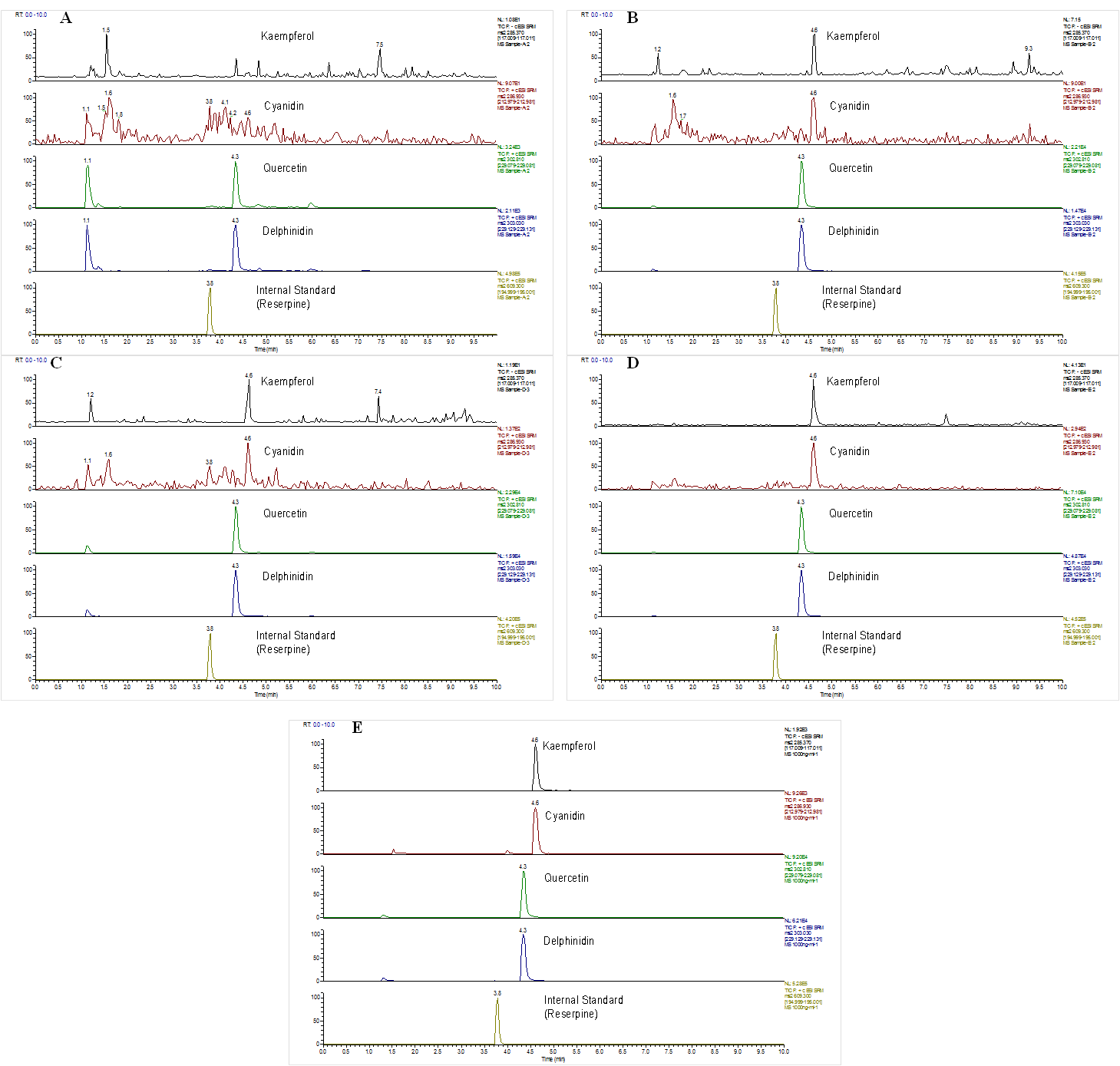
**

**Figure S8.** Analysis of flavonol and anthocyanin composition in rice seedling of transgenic and wild type treated with WBPH by using LCMS-MS.

(A-D) Extracted ion chromatograms of internal standard (kaempferol, cyanidin, quercetin and delphinidin) in wild control, wild treated, *OxF_3_H* control and *OxF_3_H* treated samples respectively.

(E) Extracted ion chromatograms of internal standard and standard compounds (kaempferol, cyanidin, quercetin, delphinidin).

**Table S1.** Gene name, accession number and primer used in present study

| **Gene name** | **Accession number** | **Primer** |
| --- | --- | --- |
| OsF3H | XM015779149.2 | caccATGGCGCCGGTGGCCA  TCACTGCTCTGACGAAGCAA |
| OsFLS | XM_015769329 | ATGGCGGAGGTGCAGAGCGTGCAG  TTACATGGGGAGCTTATTGATCTTGC |
| OsDFR | AB003496.1 | ATGGGCGAGG CGGTGAAGGG GCCAGT  ACATTTGACCAACGVTTCTGTTTC |
| OsWRKY13 | BK005016 | ATGGCGGCCGGAGAGGAGGTGATGGAT  TCAGGAGCACGGCGCGGTGGCCGCCAGGCC |
| OsSLR1 | KC611073 | ATGAAGC GCGAGTACCA AGA  CCGTCATCCGCGGCGCCG |

**Table S2.** Different medium compositions used for tissue culture

| **Medium** | **Composition** |
| --- | --- |
| Callus induction | 4.4 g/l MS powder, 30 g/l sucrose, 600 mg/l proline, 300 mg/l casein hydrolysate, 3 ppm 2,4-D, 4 g/l gerlite, 100 mg/l myo-Inositol |
| Co-cultivation | 4.4 g/l MS powder, 30 g/l sucrose, 600 mg/l proline, 300 mg/l 300 mg/l casein hydrolysate, 3 ppm 2,4-D, 4 g/l gerlite, 100 mg/l myo-Inositol, 100 mg/l acetosyringone (after autoclave), pH 5.2 |
| Selection | 4.4 g/l MS powder, 30 g/l sucrose, 30 g/l proline, 300 mg/l casein hydrolysate, 3 ppm 2,4-D, 4 g/l gerlite, 100 mg/l myo-Inositol, carbenicillin 500 mg/l, 50 mg/l spectinomycin (after autoclave), pH 5.2 |
| Regeneration | 4.4 g/l MS powder, 30 g/l sucrose, 30 g/l sorbitol, 300 mg/l casein hydrolysate, 2 mg/l kinetin, 1 mg/l NAA, 4 g/l gerlite, 100 mg/l myo-Inositol, carbenicillin 500 mg/l, 50 mg/l spectinomycin (after autoclave) |

**Table S3.** Standard equations

| **Compounds** | **Standard equation (1ng/mL – 5 µg/mL)** | **R2** | **LOD** |
| --- | --- | --- | --- |
| Quercetin | y = 0.0003x - 0.0124 | 0.9981 | 5 ng/ml |
| Kaempferol | y = 5E-06x - 0.0004 | 0.997 | 20 ng/ml |
| Delphinidin | y = 0.0002x - 0.0065 | 0.9983 | 1 ng/ml |
| Cyanidin | y = 3E-05x - 0.0029 | 0.995 | 20 ng/ml |

**Table S4. Method Validation table**

| **Name of analyte** | **Nominal Concentration (µg/ml)** | **Intra-Day** | | | | | **Inter Day** | | | | |
| --- | --- | --- | --- | --- | --- | --- | --- | --- | --- | --- | --- |
|  |  | **Concentration Measured (µg/ml)** | | | **Precision (%, RSD)** | **Accuracy (%, RE)** | **Concentration Measured (µg/ml)** | | | **Precision (%, RSD)** | **Accuracy (%, RE)** |
| Quercetin | 10 | 10.5 | ± | 0.2 | 8.5 | -5.4 | 10.9 | ± | 0.2 | 8.5 | -8.8 |
|  | 20 | 18.2 | ± | 0.7 | 6.6 | 9 | 18.6 | ± | 0.8 | 7.0 | 7.2 |
|  | 50 | 43.7 | ± | 0.9 | 8.2 | 12.5 | 44.1 | ± | 0.9 | 8.3 | 11.8 |
|  | 1000 | 1045.6 | ± | 28.6 | 0.7 | -4.6 | 1037.2 | ± | 34.2 | 5.4 | -3.7 |
|  | 5000 | 5160.8 | ± | 164.6 | 12.7 | -3.2 | 5132.3 | ± | 151.4 | 12.7 | -2.6 |
| Delphinidin | 10 | 11.4 | ± | 0.1 | 5.6 | -14.6 | 10.4 | ± | 0.1 | 5.2 | -3.8 |
|  | 20 | 19.2 | ± | 0.4 | 3.2 | 3.8 | 18.3 | ± | 0.5 | 3.3 | 8.5 |
|  | 50 | 44.7 | ± | 1.3 | 14.3 | 10.6 | 43.7 | ± | 1.3 | 14.3 | 12.6 |
|  | 1000 | 1089.8 | ± | 9.0 | 1.9 | -8.9 | 1095.8 | ± | 15.7 | 1.7 | -9.6 |
|  | 5000 | 5385.2 | ± | 199.7 | 13.8 | -7.7 | 5386.7 | ± | 200.1 | 13.8 | -7.7 |
| Cyanidin | 50 | 50.9 | ± | 0.8 | 6.7 | -1.7 | 51.1 | ± | 0.4 | 6.4 | -2.2 |
|  | 100 | 85.2 | ± | 1.9 | 5.1 | 14.8 | 85.2 | ± | 2.0 | 5.1 | 14.8 |
|  | 200 | 171.5 | ± | 3.1 | 8.7 | 14.2 | 170.4 | ± | 2.6 | 7.6 | 14.8 |
|  | 1000 | 968.2 | ± | 33.7 | 7.7 | 3.2 | 975.3 | ± | 31.3 | 6.9 | 2.5 |
|  | 5000 | 4707.6 | ± | 284 | 10.3 | 5.8 | 4707.6 | ± | 283.7 | 10.3 | 5.8 |
| Kaempferol | 50 | 54.1 | ± | 1.9 | 14.0 | -8.2 | 54.3 | ± | 1.7 | 13.3 | -8.7 |
|  | 100 | 89.3 | ± | 1.7 | 4.9 | 10.6 | 90.0 | ± | 1.8 | 4.5 | 10.0 |
|  | 500 | 427.2 | ± | 11.1 | 5.1 | 14.5 | 427.2 | ± | 11.1 | 5.1 | 14.6 |
|  | 1000 | 910.8 | ± | 10.8 | 4.9 | 8.9 | 912.3 | ± | 10.6 | 4.7 | 8.8 |
|  | 5000 | 4527.1 | ± | 198.2 | 7.3 | 9.4 | 4535.5 | ± | 194.9 | 7.3 | 9.3 |
